# Lasting DNA Damage and Aberrant DNA Repair Gene Expression Profile Are Associated with Post-Chronic Cadmium Exposure in Human Bronchial Epithelial Cells

**DOI:** 10.3390/cells8080842

**Published:** 2019-08-06

**Authors:** Heng Wee Tan, Zhan-Ling Liang, Yue Yao, Dan-Dan Wu, Hai-Ying Mo, Jiang Gu, Jen-Fu Chiu, Yan-Ming Xu, Andy T. Y. Lau

**Affiliations:** 1 Laboratory of Cancer Biology and Epigenetics, Department of Cell Biology and Genetics, Shantou University Medical College, Shantou 515041, Guangdong, China; 2 Department of Pathology and Medical Biology, University Medical Center Groningen, University of Groningen, 9713 GZ Groningen, The Netherlands; 3 GRIAC Research Institute, University Medical Center Groningen, University of Groningen, 9713 GZ Groningen, The Netherlands; 4 Provincial Key Laboratory of Infectious Diseases and Molecular Pathology, Shantou University Medical College, Shantou 515041, Guangdong, China; 5 Collaborative and Creative Center of Molecular Pathology and Personalized Medicine, Shantou University Medical College, Shantou 515041, Guangdong, China; 6 Department of Pathology and Pathophysiology, Shantou University Medical College, Shantou 515041, Guangdong, China; 7 School of Biomedical Sciences, LKS Faculty of Medicine, University of Hong Kong, Hong Kong, China

**Keywords:** cadmium, post-chronic exposure, DNA damage and repair, BEAS-2B, human lung cells, cell transformation, carcinogenesis, drug sensitivity

## Abstract

Cadmium (Cd) is a widespread environmental pollutant and carcinogen. Although the exact mechanisms of Cd-induced carcinogenesis remain unclear, previous acute/chronic Cd exposure studies have shown that Cd exerts its cytotoxic and carcinogenic effects through multiple mechanisms, including interference with the DNA repair system. However, the effects of post-chronic Cd exposure remain unknown. Here, we establish a unique post-chronic Cd-exposed human lung cell model (the “CR0” cells) and investigate the effects of post-chronic Cd exposure on the DNA repair system. We found that the CR0 cells retained Cd-resistant property even though it was grown in Cd-free culture medium for over a year. The CR0 cells had lasting DNA damage due to reduced DNA repair capacity and an aberrant DNA repair gene expression profile. A total of 12 DNA repair genes associated with post-chronic Cd exposure were identified, and they could be potential biomarkers for identifying post-chronic Cd exposure. Clinical database analysis suggests that some of the DNA repair genes play a role in lung cancer patients with different smoking histories. Generally, CR0 cells were more sensitive to chemotherapeutic (cisplatin, gemcitabine, and vinorelbine tartrate) and DNA damaging (H_2_O_2_) agents, which may represent a double-edged sword for cancer prevention and treatment. Overall, we demonstrated for the first time that the effects of post-chronic Cd exposure on human lung cells are long-lasting and different from that of acute and chronic exposures. Findings from our study unveiled a new perspective on Cd-induced carcinogenesis—the post-chronic exposure of Cd. This study encourages the field of post-exposure research which is crucial but has long been ignored.

## 1. Introduction

Cadmium (Cd), a toxic heavy metal, is a widespread environmental pollutant. Humans are commonly exposed to Cd through the consumption of contaminated food/drinking water, occupational inhalation, polluted air, and cigarette smoking. Cd is associated with many human diseases and has adverse health effects even at low concentrations [1]. It is also a well-known carcinogen where it shows a particularly strong correlation with lung cancer, one of the most common and deadliest cancers worldwide [2,3]. Although the exact mechanisms of Cd carcinogenesis remain elusive, researchers have unveiled that Cd is likely to exert its deleterious effects on cells through multiple mechanisms, including induction of oxidative stress and inflammation, inhibition of apoptosis, aberration of gene expression, alteration of DNA methylation, and interference with DNA repair system [4,5].

Cd has the ability to alter the expression of various DNA repair genes, and it is known to be able to impair DNA repair system via down-regulation of DNA repair genes expression [6,7], suppression of transcription factor activity [8], and disruption of protein’s function by binding to its zinc finger motif [4,9]. Interestingly, so far, evidence of Cd interference is overwhelmingly available only for the repair pathways of single-stranded DNA (ssDNA) damage (e.g., base excision repair (BER), nucleotide excision repair (NER), and mismatch repair (MMR)), but very limited for double-stranded DNA (dsDNA) damage such as homologous recombinational repair (HRR) and non-homologous end-joining (NHEJ). In order to better understand the mechanisms of Cd carcinogenesis, one must look at the impacts of Cd on the overall DNA repair systems instead of a single DNA repair enzyme. Also, almost all previous studies on Cd-induced toxicity and carcinogenicity focused only on examining the effects of either acute [10,11,12] or chronic [13,14,15] Cd exposure but have neglected the potential implications of post-chronic Cd exposure on human health.

The term “chronic exposure” is defined as continuous exposure to a particular compound over an extended period of time, whereas “post-chronic exposure” is referred to as the state after chronic exposure, when it is no longer exposed to the particular compound. In the era where exposure to Cd is of a growing environmental health concern, systematic research on post-chronic Cd exposure can be extremely important since post-chronic Cd exposure is not only especially relevant to people like ex-smokers and retired workers in high-risk occupations, but also to people from the general population who live in Cd-polluted environments [16,17]. Unfortunately, research on post-chronic exposure of Cd (and other toxic compounds) has so far remained largely unexplored, and this may be in part due to the lack of such experimental models.

Recent studies have revealed the epigenotoxicity of Cd and thus indicated that Cd toxicity might be heritable and able to last for generations [18,19,20]. These findings reinforce the need for research on post-chronic Cd exposure. In the current study, we established a unique post-chronic Cd-exposed human bronchial epithelium BEAS-2B cell model (the “CR0” cells) and used this cell model to study the possible long-term carcinogenic effects of post-chronic Cd exposure on human lung cells. We specifically focused on the aspects of DNA damage and repair by measuring the DNA repair capacity and systematically assessing the transcriptomic profile of DNA repair genes in the CR0 cells. Furthermore, the clinical relevance of our findings in relation to lung cancer and cigarette smoking was evaluated. Lastly, we tested the chemotherapeutic drug susceptibility of CR0 cells in order to lay a foundation for future direction on chemo-intervention against Cd-induced lung cancer. Overall, our study unveiled a new perspective on Cd-induced carcinogenicity—the post-chronic exposure of Cd.

## 2. Materials and Methods

### 2.1. Establishment of Chronic and Post-Chronic Cd-Exposed Human Bronchial Epithelial BEAS-2B Cell Models

The BEAS-2B cell line was purchased from the American Type Culture Collection (ATCC) (Rockville, MD, USA). Short Tandem Repeat (STR) profiling was performed for cell line authentication by Guangzhou Cellcook Biotech Co., Ltd. (Guangzhou, China; the STR report is available upon request). BEAS-2B cells were isolated from normal human bronchial epithelium obtained from autopsy of a non-cancerous individual. Cells were routinely grown in LHC-9 medium (Gibco, New York, NY, USA) at 37 °C in an atmosphere of 5% CO_2_/95% air as recommended by the ATCC. LHC-9 is a defined, serum-free medium, which is prepared by mixing LHC basal medium with growth factors, cytokines, and supplements as described previously [21]. 

For the establishment of a chronic Cd-exposed cell line, BEAS-2B cells were stepwise-adapted to environmentally-relevant concentrations of CdCl_2_ in LHC-9 medium with concentrations ranging from 1 to 20 µM after approximately 20 passages, and subsequently cultured in the presence of 20 μM CdCl_2_ for at least 3 months as described previously [19]. Sham-exposed BEAS-2B cells were obtained as passage-matched control cells (the “PM” cells). The resulting Cd-resistant BEAS-2B cells, along with the PM control cells, were then continuously cultured in Cd-free LHC-9 medium for more than a year to obtain the post-chronic Cd-resistant BEAS-2B cell line (the “CR0” cells).

### 2.2. Determination of Cell Proliferation and Cell Viability

CR0 and PM cells were seeded in 96-well plates at 2000–2500 cells per well in LHC-9 medium and incubated for at least 8 h. The medium was then changed, and the cells were treated with various concentrations of cisplatin (Hansoh Pharmaceutical, Jiangsu, China), gemcitabine (Selleck, Shanghai, China), vinorelbine tartrate (Selleck, Shanghai, China), or H_2_O_2_ (Sinopharm Chemical Reagent Co., Shanghai, China). Cells were incubated for a designated period of time, and at the end of the experiments, cell proliferation or cell viability was measured by 3-(4,5-dimethylthiazol-2-yl)-5-(3-carboxymethoxyphenyl)-2-(4-sulfophenyl)-2H-tetrazolium (MTS) assay according to manufacturer’s protocol (Promega, Madison, WI, USA).

### 2.3. Detection of DNA Damage and DNA Repair Capacity

DNA damages and DNA repair capacity were determined by single cell gel electrophoresis (the “comet assay”). An alkaline comet assay was performed according to Olive and Banáth, with minor modifications and adjustments on the glass slides and agarose concentrations for better agarose adhesion [22]. Images of at least 50 non-overlapping cells per sample were captured using a fluorescence microscope (Axiovert 40 CFL, ZEISS, Shanghai, China) attached with a digital camera (MicroPublisher 5.0 RTV, Teledyne QImaging, Surrey, British Columbia, Canada) and viewed by the accompanying software QCapture (v2.9.13, Quantitative Imaging Corp.). Experiments were performed at least three times for each sample. Individual images were analyzed using the Comet Assay Software Project software (v1.2.3b2, CASPLab), and the means of the % tail DNA and tail extent moment from three separate experiments were calculated as measures of DNA damage. The tail extent moment is defined as “% tail DNA × comet tail length ÷ 100”.

### 2.4. Determination of Colony Formation Ability

Colony (focus) formation assay was performed to assess the cells’ ability to form colonies as described in Alvarez et al. with minor modifications [23]. Briefly, cells were seeded in 6-well plates at 500 cells per well in LHC-9 medium with 20 μM CdCl_2_ and cultured for a total of seven days. Cell foci were stained with crystal violet and images were captured using a digital scanner (V370 Photo, EPSON, Long Beach, CA, USA). Stained foci were solubilized and extracted using 10% acetic acid and absorbance was measured at 595 nm.

### 2.5. Detection of Histone H2AFX and γH2AFX Expression

Acid extraction of histones from PM and CR0 cells was performed as described previously [24]. For the detection of H2AFX and γH2AFX expression, 1 μg of histone was separated using 15% SDS-PAGE and transferred to polyvinylidene difluoride membranes (Merck-Millipore, Darmstadt, Germany). The membrane was blocked with 5% (*w/v*) non-fat milk in tris-buffered saline containing 0.05% Tween-20 for 2 h and incubated with H2AFX (1:1000, A11361, ABClonal), γH2AFX (1:1000, AP0099, ABClonal), or H3 (1:2000, 4499, CST) antibody at 4 °C overnight, followed by anti-rabbit secondary antibody (1:5000, sc-2004, Santa Cruz), and detected by chemiluminescence (Tanon5200, Tanon Science & Technology Co., Ltd, Shanghai, China).

### 2.6. Assessment of Global DNA Repair-Related Gene Expression Profile

The relative DNA repair-related gene expression of CR0 over PM control cells was analyzed using real-time quantitative polymerase chain reaction (RT-qPCR) on an ABI7500 Real-Time PCR System (Applied Biosystems, Foster City, CA, USA), and calculated using the comparative C_T_ method (2^-ΔΔC^_T_ method) [25]. Total RNA was extracted with QIAzol^®^ Lysis Reagent followed by RNeasy Mini Kit (Qiagen-SABiosciences, Germantown, MD, USA) and reverse-transcribed to cDNA by RT^2^ First Strand Kit (Qiagen-SABiosciences, Germantown, MD, USA) according to the manufacturer’s instructions. For RT-qPCR, cDNA template derived from 10 ng of total RNA was amplified by the appropriate primer set in a reaction containing RT^2^ SYBR^®^ Green ROX qPCR Mastermix (Qiagen-SABiosciences, Germantown, MD, USA). 

A total of 117 DNA repair-related genes involved in 16 different DNA repair pathways were examined in this study (summarized in Table 1). Among them, 78 genes were evaluated using RT^2^ Profiler™ PCR Arrays for Human DNA Repair (Qiagen-SABiosciences, Germantown, MD, USA) while 39 genes were evaluated based on primer sequences obtained from RTPrimerDB database (www.rtprimerdb.org) [26] or designed using Primer-BLAST (Primer3 and NCBI, Bethesda, MD, USA). Here, we used a novel “scoring system” involving the use of multiple reference/housekeeping genes to provide us a less biased method to analyze gene expression data. These reference genes are β-actin (*ACTB*), β-2-microglobulin (*B2M*), glyceraldehyde-3-phosphate dehydrogenase (*GAPDH*), hypoxanthine-guanine phosphoribosyltransferase (*HPRT1*), 60S acidic ribosomal protein P0 (*PRLP0*), and “5HKGs” (5-housekeeping genes; the “expression value” of 5HKGs is created based on the average expression levels of the above five reference genes. The specificity and PCR amplification efficiency of the designed primers were determined by melt curve analysis and a standard curve plot, respectively. All primers used had a single defined melt curve peak and an efficiency of 90%–110%. Information for primers used in this study is listed in Table S1.

### 2.7. Analysis of Clinical Data Obtained from Online Databases

Expression of selected DNA repair genes in lung cancers was determined through analysis in the cBioPortal database (www.cbioportal.org) [27]. Two clinical datasets of different types of lung cancer, lung adenocarcinoma (517 samples), and lung squamous cell carcinoma (501 samples), were obtained from The Cancer Genome Atlas (TCGA) database and analyzed. The mRNA expression z-score threshold was set at ± 1.5; only genes with expression fold-change greater than 1.5 between the tumor and normal samples were considered significant. The z-score indicates the relative expression of an individual gene in the tumor sample to the gene’s expression distribution in a reference population (normal samples), and it is calculated as ((expression in tumor sample – mean expression in reference sample) ÷ standard deviation of expression in reference sample).

### 2.8. Statistical Analysis

Statistical analysis was performed using the GraphPad Prism^®^ 6 software (v6.02, GraphPad Software Inc.). All values in the bar charts and line charts are expressed as mean ± standard deviation (SD). All experiments were performed at least three times, and they showed similar trends in their results. Results from one representative experiment were shown unless mentioned otherwise. Two-tailed Student’s *t*-test was used to determine significant differences between the means of analyzed data unless mentioned otherwise. *p* ≤ 0.05 was considered statistically significant.

## 3. Results

### 3.1. Establishment of a Post-Chronic Cd-Exposed BEAS-2B Cell Line (the “CR0” Cells)

**Figure 1:**
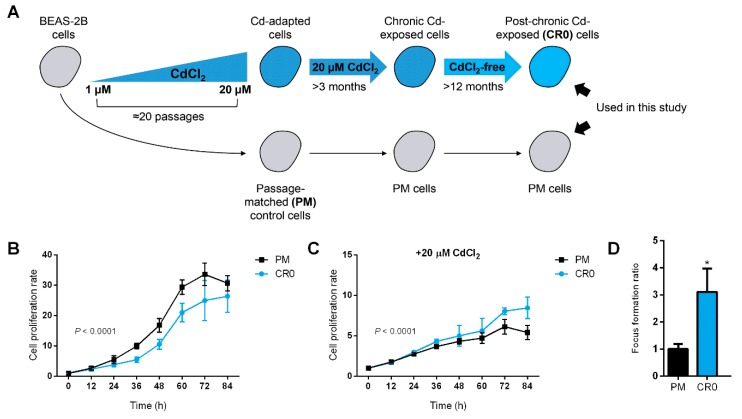
Establishment of a post-chronic Cd-exposed human lung cell line and evaluation of its Cd-resistance and DNA damage and repair capacity. (**A**) Schematic of the establishment of chronic and post-chronic Cd-exposed BEAS-2B cell line used in the previous [19] and current study; the post-chronic Cd-exposed BEAS-2B cells are termed as “CR0” while the passage-matched control cells are termed as “PM”. (**B**,**C**) Cell proliferation rate of CR0 and PM cells grown in standard LHC-9 medium or culture medium containing 20 µM CdCl_2_ was measured by 3-(4,5-dimethylthiazol-2-yl)-5-(3-carboxymethoxyphenyl)-2-(4-sulfophenyl)-2H-tetrazolium (MTS) assay. Results are representative of at least three independent experiments, and error bars represent mean ± SD of at least three technical replicates. (**D**) Colony formation assay of PM and CR0 cells treated with 20 µM of CdCl_2_ and grown for 7–10 days.

Previously, we established a transformed BEAS-2B cell line which was stepwise-adapted to environmentally-relevant concentrations of CdCl_2_ (1–20 µM) [19]. Cells from this cell line exhibited transformed cell phenotypes as evidenced by their ability to anchorage-independent growth on soft agar and enhanced cell migration. We also found that Cd could induce epigenotoxicity in the BEAS-2B cells as the levels of several important histone post-translational modification (PTM) marks, including those involved in DNA repair, were significantly altered upon chronic Cd exposure. These results indicated that Cd could exert its toxic effects to the cellular epigenetic circuit in which the Cd-induced toxicity might be heritable. Here, we established a post-chronic Cd-exposed cell line by culturing the Cd-transformed BEAS-2B cells in Cd-free condition for at least 12 months (Figure 1A). The post-chronic Cd-exposed BEAS-2B cells are termed as “CR0” while the passage-matched control cells are termed as “PM”, and they are used in the current study as unique cell models to study the effects of post-chronic Cd exposure on DNA repair system in human lung cells.

### 3.2. Cd-Resistant Cells Retain Cd Tolerance Feature for an Extended Period of Time

We compared the growth rates of both CR0 and PM cells and discovered that CR0 cells had decreased cell proliferation when cultured in normal Cd-free growth medium (Figure 1B). However, when cultured in medium supplied with 20 µM of CdCl_2_, the CR0 cells showed greater cell proliferation than the PM control cells, although an overall reduction in growth rate was observed in both CR0 and PM cells (Figure 1C). In addition, colony formation assay revealed that CR0 cells were able to form significantly more colonies than the PM control cells (Figure 1D; Figure S1A). These results indicate that CR0 cells are still able to tolerate Cd to some extent. However, the tolerance level of CR0 cells is lower as compared to our previously established transformed BEAS-2B cells that were still maintained in 20 µM CdCl_2_ at the time of experiment, suggesting the potential of Cd-resistant cell lines to lose their Cd resistance trait over time when grown in a Cd-free condition for an extended period of time (Figure S1B).

### 3.3. Exposure to Cd Resulted in Cells Having Lasting DNA Damage and Reduced DNA Repair Capacity

**Figure 2:**
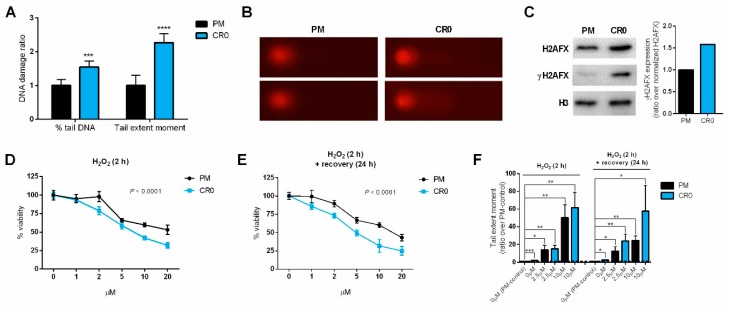
Evaluation of DNA damage and repair capacity in the post-chronic Cd-exposed cell line. (**A**) Intrinsic DNA damage level of CR0 cells (over PM control cells) was measured by comet assay (*n* = 6); two DNA damage parameters, % tail DNA and tail extent moment, are shown. (**B**) Representative images of comet assay in PM and CR0 cells stained with 5 μg/mL of propidium iodide after gel electrophoresis. (**C**) Expression of histone H2AFX and phosphorylated H2AFX (γH2AFX) were determined by Western blot (see Figure S2 for full blot). (**D**,**E**) Cell viability of PM and CR0 cells exposed to various concentrations of DNA damage-inducing agent H_2_O_2_ for 2 h without or with a 24 h recovery period was measured by MTS assay; results were representative of at least three independent experiments, and error bars represent mean ± SD of at least three technical replicates. (**F**) Tail extent moment of PM and CR0 cells exposed to 0, 2.5, and 10 µM of H_2_O_2_ for 2 h without or with a 24 h recovery period was analyzed by comet assay. * *p* ≤ 0.05; ** *p* ≤ 0.01; *** *p* ≤ 0.001.

It is well-documented that acute/chronic Cd exposure can disrupt cellular DNA repair system, but the effects of post-chronic Cd exposure remain elusive. Here, we checked the native/intrinsic DNA damage in CR0 cells using the comet assay and revealed that when compared to PM cells, the CR0 cells possess greater DNA damage as shown by higher % tail DNA and tail extent moment values (Figure 2A,B). We then assessed the expression of histone γH2AFX (a well-known DNA damage indicator) in the CR0 cells and found that CR0 cells have higher H2AFX and γH2AFX expressions (Figure 2C; for full blot see Figure S2). This result again reinforced that CR0 cells have higher intrinsic DNA damage as phosphorylation of histone H2AFX (γH2AFX) is known to be highly correlated with DNA damage and repair events in the cells [28].

To assess the DNA repair capacity of the CR0 cells, we exposed the cells to H_2_O_2_, a DNA damaging agent that induces ssDNA breaks, and measured their cell viability and DNA damage upon direct or post (24 h recovery time) H_2_O_2_ exposure. The cell survivability test indicated that CR0 cells are generally more sensitive to H_2_O_2_ than the PM cells (Figure 2D,E). In terms of DNA damage, both PM and CR0 cells show very high DNA damages when exposed to H_2_O_2_ for 2 h (Figure 2F). This was an expected result since H_2_O_2_ is known for inducing severe DNA damage and is often used as a positive control in comet assays [22]. Although no significant differences were observed due to large error bars, it appeared that CR0 cells might have greater DNA damages than the PM cells upon H_2_O_2_ exposure, which was more obvious after a 24 h recovery period (Figure 2F). Taken together, these results not only show that the CR0 cells have a lower DNA repair capacity, but also indicates that they were likely to be more susceptible to DNA damage-inducing agents.

### 3.4. Aberrant Expression of DNA Repair Gene Profile Is Associated with Post-Chronic Cd Exposure

**Figure 3:**
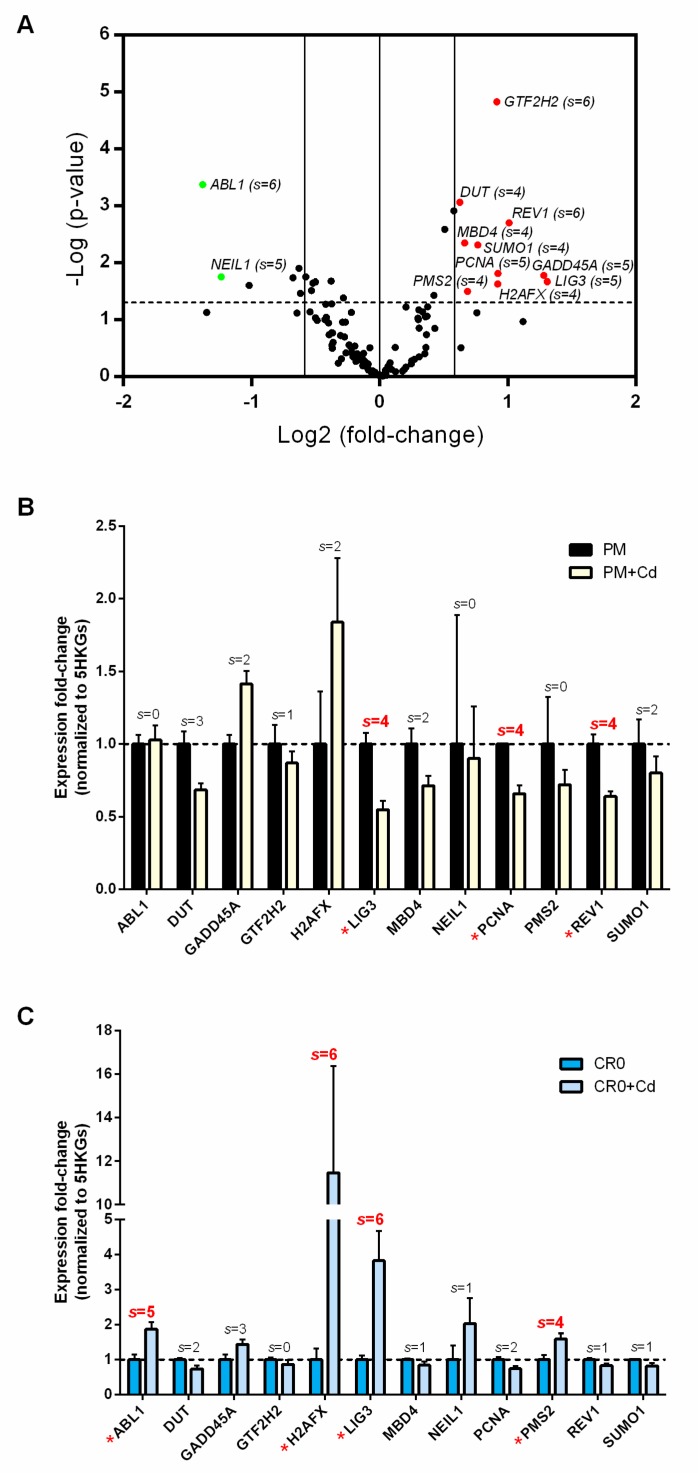
DNA repair gene expression profile associated with Cd exposure. (**A**) Volcano plot of 117 DNA repair gene expression profiles of CR0 over PM cells when normalized to 5-housekeeping genes (5HKGs), modified from Figure S3F. Only expression of genes with an *s*-score (*s*) ≥ 4 were considered as significantly altered and highlighted in green (down-regulated) or red (up-regulated). Specifically, to gain an *s*-score (*s* = 1), a gene has to be *p* ≤ 0.05 and fold-change ≥1.5 upon normalization to a selected reference gene. Six references genes (*ACTB*, *B2M*, *GAPDH*, *HPRT1*, *PRLP0*, and 5HKGs) were used. Vertical lines on the *x*-axis represent 1.5 or –1.5 fold-change; dash line on the *y*-axis represents *p* ≤ 0.05. (**B**,**C**) Expression of the 12 differentially expressed DNA repair genes in PM and CR0 cells treated with 20 µM of CdCl_2_ for 72 h. Only expressions of genes with *s* ≥ 4 (highlighted in red asterisk) were considered significantly altered.

DNA repair is a complicated process that involves a network of multiple pathways and hundreds of genes. Here, we screened the expression of 117 DNA repair-related genes involved in 16 different DNA repair pathways in the PM and CR0 cells (see Table 1). Here, we developed a “scoring system” in order to more accurately assess the DNA repair gene expression profile data. Unlike the conventional method where only one reference/housekeeping gene is used, in our scoring system, multiple reference genes were utilized to normalize the expression of the analyzed genes. Specifically, each DNA repair gene was individually normalized to six different reference genes: *ACTB*, *B2M*, *GAPDH*, *HPRT1*, *PRLP0*, and “5HKGs”, and a significance-score (*s*-score = 1) was given when a gene showed significant results (*p* ≤ 0.05 and fold-change ≥1.5) upon normalization to a reference gene (Figure S3A–F; Table S2). Out of the 117 DNA repair genes, 52 genes were shown to be significantly up- or down-regulated in the PM vs. CR0 cells when normalized to either one of the six reference genes (with *s*-score ≥ 1; Figure S4A). However, to diminish false-positive results, we only considered genes with *s*-score ≥ 4 to be truly significant. Based on this requirement, only 12 genes qualified, as shown in Figure 3A, in which 10 genes were up-regulated (*DUT*, *GADD45A*, *GTF2H2*, *H2AFX*, *LIG3*, *MBD4*, *PCNA*, *PMS2*, *REV1*, and *SUMO1*) and only two were down-regulated (*ABL1* and *NEIL1*) (Figure S4B). 

Unlike previous studies where most DNA repair genes were reported to be suppressed upon acute or chronic Cd exposure [4,6,7,8,13,29,30], up-regulation of 10 out of 12 DNA repair genes in this study suggests that acute/chronic and post-chronic Cd-exposed cells have different transcriptomic landscapes of DNA repair. Most importantly, the DNA repair transcriptome of the CR0 cells also suggests that chronic Cd exposure has long-term effects on the expression of DNA repair-related genes in human lung cells.

The 12 differentially expressed DNA repair genes identified in our study are recognized to be involved in a diverse range of DNA repair pathways (Figure S4C). However, it appears that Cd may selectively have a greater influence on certain DNA repair pathways. Taking the five major repair pathways for example, five (*GADD45A*, *LIG3*, *MBD4*, *PCNA*, and *NEIL1*), four (*GADD45A*, *GTF2H2*, *LIG3*, and *PCNA*), and three (*PCNA*, *PMS2*, and *ABL1*) genes were involved in the major ssDNA damage repair: BER, NER, and MMR, respectively. However, as for the dsDNA damage repair, only four (*GADD45A*, *H2AFX*, *LIG3*, and *REV1*) genes were involved in the HRR and none for the NHEJ. These findings are remarkably similar with available literature where the evidence for Cd interference in BER, NER, and MMR is overwhelming, but not much on the repair of dsDNA breaks involving HRR and NHEJ [4,5,6]. 

Furthermore, some of the DNA repair genes investigated in this study were selected for immunoblotting analysis (Figure S5). Results indicated that the protein expressions of most of the selected genes, including *H2AFX*, *LIG3*, *PMS2*, and *NEIL1*, were largely in agreement with their gene expression profiles. In summary, this is the first report to show that the expression of a panel of DNA repair genes was altered in response to post-chronic Cd exposure.

### 3.5. Short-Term Cd Exposure Reveals Different DNA Repair Transcriptomic Landscapes in Normal and Cd-Exposed Cells

We then evaluated the effects of short-term Cd exposure on the expression of the 12 differentially expressed DNA repair genes identified in the current study by treating both PM and CR0 cells with 20 µM of CdCl_2_ for 72 h. Interestingly, results indicated that most of the genes were down-regulated in the PM cells (Figure 3B) but up-regulated in the CR0 cells (Figure 3C). Specifically, three genes (*LIG3*, *PCNA*, and *REV1*) were significantly down-regulated in the PM cells whereas four genes (*ABL1*, *H2AFX*, *LIG3*, and *PMS2*) were significantly up-regulated in the CR0 cells. For the PM cells, only two genes, *GADD45A* and *H2AFX*, appeared to be up-regulated but the expression changes were not considered significant based on our criteria for analyzing the gene expression data (the *s*-score is not ≥4; Table S3). Overall, these results again indicate that the normal (PM) and Cd-transformed (CR0) lung cells have different transcriptomic landscapes of DNA repair and suggest that both cell lines would likely react differently towards environmental stimuli such as Cd.

### 3.6. Clinical Database Analysis of Lung Cancer Samples with Different Smoking Histories

A reduced DNA repair ability or an impaired DNA repair system can cause a cell to accumulate DNA damages and mutations, which may eventually become malignant. Thus, cells exposed to Cd have the potential to become cancerous. Here, we utilized online clinical datasets (TCGA, provisional) to analyze the expression of the 12 differentially expressed DNA repair genes in lung cancer patients with lung adenocarcinoma (517 samples) or lung squamous cell carcinoma (501 samples). We found that the expressions of these 12 genes were significantly altered (mRNA expression z-score threshold ± 1.5) in 6%–16% of lung adenocarcinoma patients (31–83 out of 517 patients) (Figure S6A) and in 2.8%–36% of lung squamous cell carcinoma patients (14–180 out of 501 patients) (Figure S6B), depending on the gene analyzed. Interestingly, the majority of these DNA repair genes, with the exception of *ABL1*, tend to be up-regulated in their expressions (*ABL1* is also down-regulated in the CR0 cells). These results are highly similar to the DNA repair gene expression profile of the CR0 cells, where only *NEIL1* was the one gene that did not match (down-regulated in CR0 cells but up-regulated in lung cancer patients analyzed). Thus, these findings indicate that the expression profile of the DNA repair genes identified in our study may possibly be used to define abnormal lung cells and suggest that the Cd-transformed CR0 cells may be cancerous, also as supported by our previous study [19].

**Figure 4:**
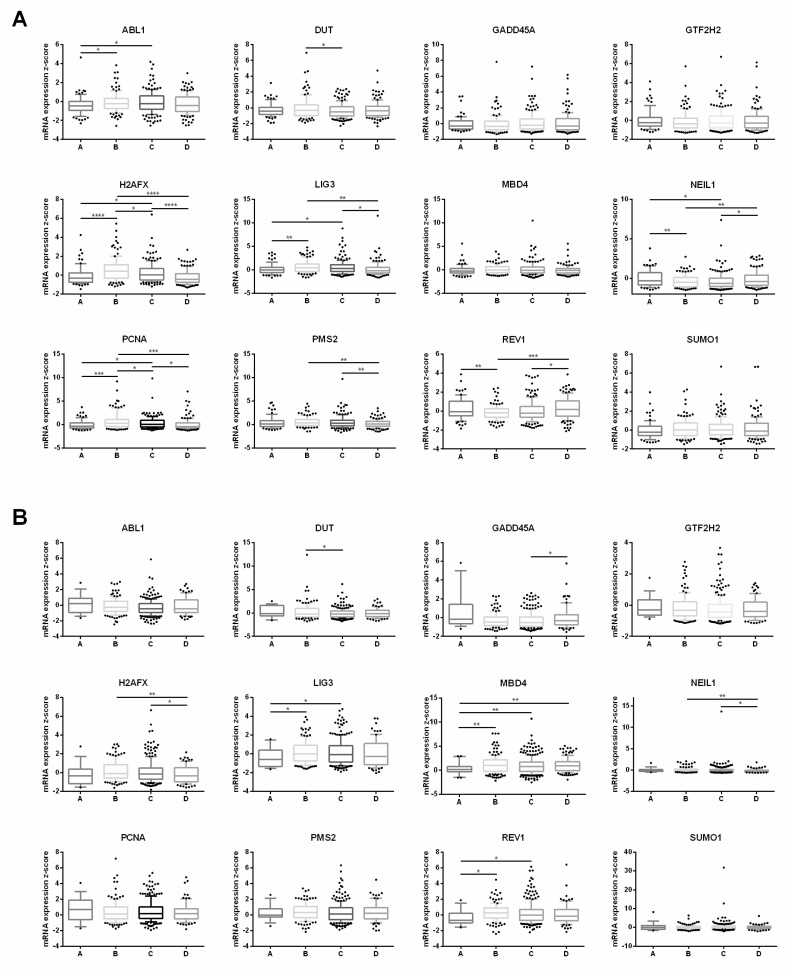
Box and whisker plots of the expression of 12 differentially expressed DNA repair genes associated with post-chronic Cd exposure. Two datasets, (**A**) lung adenocarcinoma samples and (**B**) lung squamous cell carcinoma samples with different smoking history, were analyzed. Data were derived from The Cancer Genome Atlas (TCGA, provisional) and sorted using cBioPortal database. The box and whiskers plots are plotted in the style of 10–95 percentile. Smoking status: A = lifelong non-smoker (76 samples for lung adenocarcinoma; 18 samples for lung squamous cell carcinoma samples); B = current smoker (119 samples; 133 samples); C = current reformed smoker for ≤15 years (135 samples; 250 samples); D = current reformed smoker for >15 years (169 samples; 83 samples). Two-tailed Student’s *t*-test was used to determine significant differences between the means of the groups. *p* ≤ 0.05 was considered statistically significant. * *p* ≤ 0.05; ** *p* ≤ 0.01; *** *p* ≤ 0.001; **** *p* ≤ 0.0001.

Since cigarette smoking is one of the primary sources of Cd exposure in humans [17,31], we utilized the same clinical datasets and analyzed the expression of the 12 genes based on different patient smoking history categories: lifelong non-smoker, current smoker, current reformed smoker for ≤15 years, and current reformed smoker for >15 years (Figure 4). It was shown that the lung adenocarcinoma dataset (Figure 4A) was more variable in gene expression than the lung squamous cell carcinoma dataset (Figure 4B). The above data are also summarized in Table S4. For lung adenocarcinoma, four genes (*H2AFX*, *LIG3*, *NEIL1*, and *PCNA*) in the “non-smoker vs. smoker” and “non-smoker vs. ex-smoker (≤15 years)” categories show the same gene expression patterns as the PM versus the CR0 cells: up-regulated *H2AFX*, *LIG3*, and *PCNA* and down-regulated *NEIL1*. However, the expressions of these four genes are all becoming opposite in “smoker vs. ex-smoker (≤15 years)”, “smoker vs. ex-smoker (>15 years)”, and “ex-smoker (≤15 years) vs. ex-smoker (>15 years)”. In addition, *LIG3* is the only gene that shows the same expression trend in “non-smoker vs. smoker” and “non-smoker vs. ex-smoker (≤15 years)” in both datasets. Overall, these results showed that smoking might play a role in the expression of some of these DNA repair genes in lung cancer patients, particularly in patients with lung adenocarcinoma.

### 3.7. Post-Chronic Cd Exposure Resulted in Cells Having Reduced DNA Repair Capacity and More Susceptible to Chemotherapy

**Figure 5:**
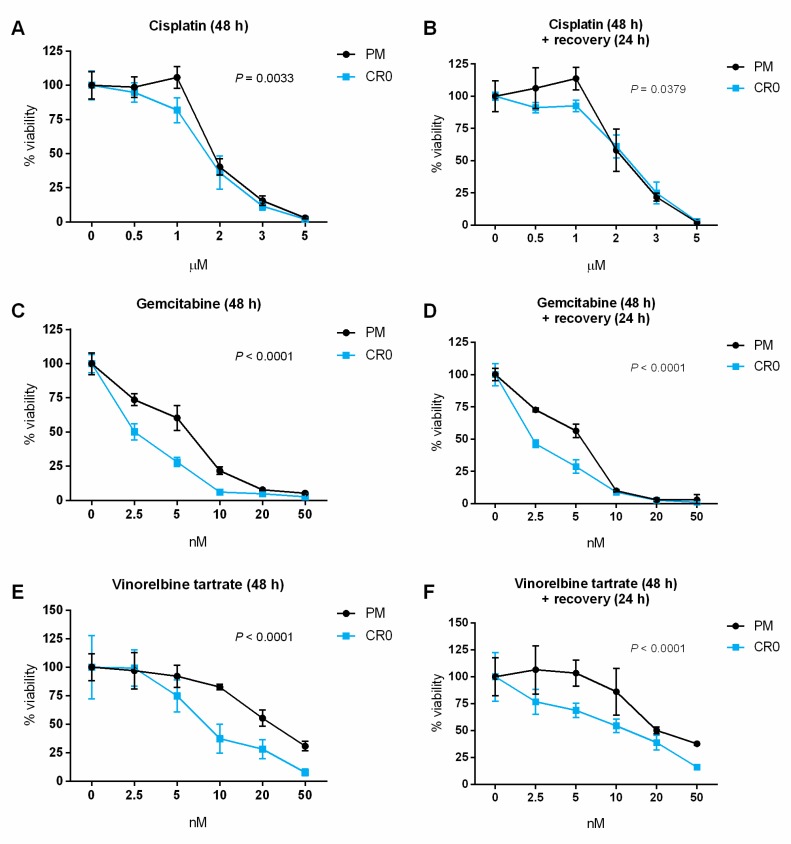
Cell viability of PM and CR0 cells exposed to selected chemotherapeutic drugs. Three drugs were tested: (**A**,**B**) cisplatin, (**C**,**D**) gemcitabine, and (**E**,**F**) vinorelbine tartrate. Cells were treated with various concentrations of the above chemotherapeutic drugs for 48 h and their viability was measured by MTS assay (A,C,E) directly or (B,D,F) after a 24 h recovery period. Results are representative of at least three independent experiments with a similar survival trend. The percentage of viability was plotted as 100% for untreated CR0 or PM controls. Error bars represent mean ± SD of at least four technical replicates. Two-way ANOVA was used to determine significant differences between CR0 and PM by comparing the means at each concentration point, and *p* ≤ 0.05 was considered statistically significant.

There is currently no information on how post chronic Cd-transformed cells would react to anticancer treatment. Taking advantage of our established post-chronic Cd-exposed CR0 cells, we investigated the sensitivity of this cell line against three chemotherapeutic drugs: cisplatin, gemcitabine, and vinorelbine tartrate. Here, PM and CR0 cells were correspondingly treated with various concentrations of the above chemotherapeutic drugs for 48 h and their viability was measured directly or after a 24 h recovery period. Results show that CR0 cells were generally more sensitive to all the drugs tested than the PM control cells (Figure 5A–F). 

**Figure 6:**
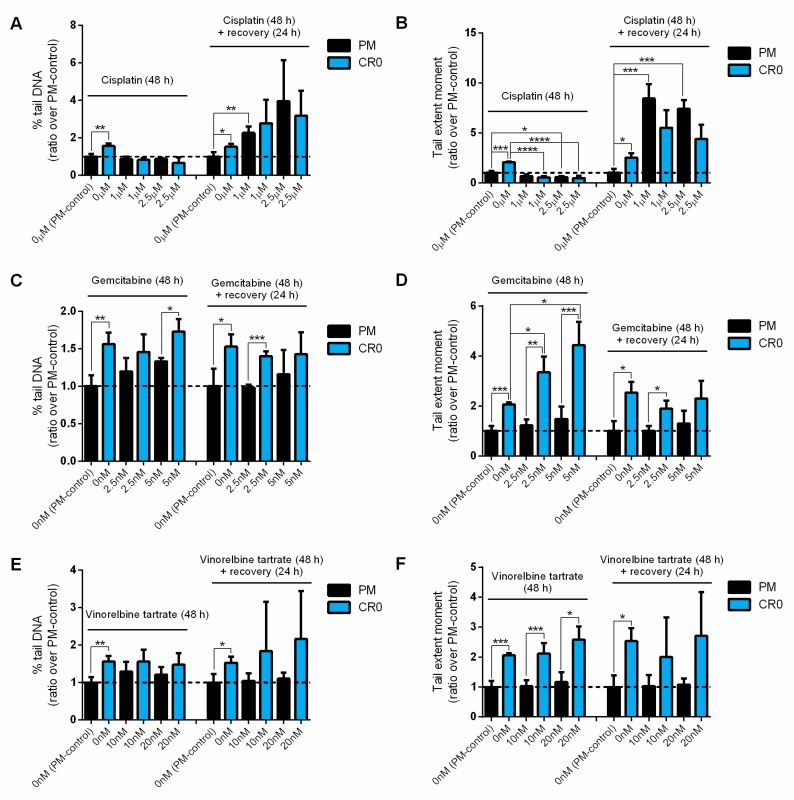
DNA damage and repair capacity of PM and CR0 cells exposed to selected chemotherapeutic drugs. Three drugs were tested: (**A**,**B**) cisplatin, (**C**,**D**) gemcitabine, and (**E**,**F**) vinorelbine tartrate. Cells were treated with two selected concentrations of the above chemotherapeutic drugs for 48 h and their DNA damages were measured by comet assay directly or after a 24 h recovery period. Two DNA damage parameters are shown: (A,C,E) % tail DNA and (B,D,F) tail extent moment. Results shown are ratio over untreated PM-control. Error bars represent mean ± SD of three separate experiments. Dash line on the *y*-axis represents ratio value of PM-control at one.

We also assessed the DNA damage and repair capacity of the PM and CR0 cells when exposed to cisplatin, gemcitabine, and vinorelbine tartrate via comet assays. Under normal circumstances, increased % tail DNA and tail extent moment indicate greater DNA damages and/or reduced DNA repair ability. However, both PM and CR0 cells showed slightly reduced % tail DNA and tail extent moment when exposed to cisplatin but showed increased % tail DNA and tail extent moment after a 24 h recovery period due to the cisplatin’s mechanism of action (Figure 6A,B). Cisplatin binds to DNA to form intra- and inter-strand crosslinks and thereby, inhibits DNA replication [32]. The crosslinks between cisplatin and DNA cannot readily be unwound by the alkaline comet assay, resulting in decreased DNA electrophoretic mobility [33]. Hence, the decrease in tail extent moment is indicative of crosslink formation, whereas the increase in tail extent moment after a recovery period indicates crosslink repair (Figure 6B). 

When exposed to gemcitabine and vinorelbine tartrate, CR0 cells generally showed greater DNA damage and reduced repair capacity (Figure 6C–F). Results indicated that the % tail DNA and tail extent moment of the CR0 cells were always greater than the PM cells when treated with the same concentration of gemcitabine or vinorelbine tartrate. Also, CR0 cells showed significant increases in tail extent moment when treated with increased concentrations of gemcitabine at 48 h but the PM cells maintained relatively the same (Figure 6D). These results again show that CR0 cells have reduced DNA repair capacity and they are much more vulnerable to the chemotherapeutic drugs tested. Overall, the above findings suggest that Cd-induced lung cancer could be more effectively treated with chemotherapy.

## 4. Discussion

To the best of our knowledge, this is the first study to investigate the long-term effects of post-chronic Cd exposure in human lung cells. This study is also the first to systematically examine the expression of DNA repair genes upon post-chronic Cd exposure on a large-scale. Cd is a cumulative toxin of increasing environmental and occupational concern because of its long biological half-life [34]. Cd occurs naturally in the living environment and has been widely used in the field of industry since the beginning of the 1940s [35]. Although the exact molecular mechanisms are not yet fully understood, the potential of acute and chronic Cd exposure to cause cytotoxicity and carcinogenicity has been documented in many experimental and epidemiological studies [10,11,12,13,14,15]. However, knowledge regarding post-chronic Cd exposure is lacking. One of the main challenges in the post Cd (or other carcinogens) exposure-related research is the lack of such cell models.

In regard to human subjects, it is extremely difficult to define samples that are only exposed to Cd since the exposure to environmental pollutants is often multifaceted and multifactorial, and it is likely to result in complex toxic interactions in the body [36]. Therefore, in order to understand the mechanisms underlying cytotoxic or carcinogenic effects of Cd in humans, establishments of long-term and immortalized human lung epithelial cell cultures or lung tissue from animal models are essential [3]. In the current study, we established a post Cd-exposed human bronchial epithelial BEAS-2B cell line (the CR0 cells) to study the effects of post-chronic Cd exposure on DNA damage and repair.

The CR0 cells were established from the BEAS-2B cells because previous studies have shown that different organs exhibit a different Cd absorption rate, and lung tissue is one of the main targets of Cd toxicity [2,37,38]. It was estimated that more than 50% of Cd could be absorbed by the lungs whereas only less than 10% of ingested Cd was absorbed in the gastrointestinal system [37]. This may help explain why the most obvious correlation between Cd and human diseases is found in the lungs [39]. Additionally, Cd is a well-known carcinogen where it particularly shows a strong correlation with lung cancer [40]. In fact, Cd has been classified as a top human carcinogen by international organizations such as the World Health Organization’s International Agency for Research on Cancer [41].

Previously, the expression of many important DNA repair genes (e.g., *CRY1*, *ERCC1*, *LIG4*, *MLH1*, *MSH2*, *MSH6*, *MSH7*, *OGG1*, *PCNA*, *RAD21*, *RAD50*, *XRCC1*, and *XRCC4*) in human, animal, or plant cells were found to be suppressed by Cd [6,8,13,29,30]. In Zhou et al., the overexpression of DNA methyltransferase genes (*DNMT1* and *DNMT3a*) resulted in the silencing of DNA repair genes (*MSH2*, *ERCC1*, *XRCC1*, and *OGG1*) in CdCl_2_-transformed human bronchial epithelial 16HBE cells and tumorigenic cells isolated from a xenograft nude mouse model [7]. However, the expression of most of the abovementioned genes from previous studies was not differentially expressed in the CR0 cells in the current study. Here, the differently expressed DNA repair genes identified in the CR0 cells were mostly up-regulated (ten genes up and only two down), and this may be due to the lasting DNA damage found in the cells because the cells require more DNA repair proteins to cope with the damages. These findings indicate that acute/chronic and post-chronic Cd exposures resulted in different transcriptomic landscapes of DNA repair.

Expression of several DNA repair genes in CR0 cells and control PM cells also appeared to be different upon short-term Cd exposure: down-regulated in the PM cells but up-regulated in the CR0 cells. These results again indicated that both cell lines had different transcriptomic landscapes and would likely react differently towards environmental stimuli. The up-regulation of DNA repair gene expression in the CR0 cells is certainly an interesting and distinctive feature of the post-chronic Cd-exposed cell line and may be used as biomarkers for identifying post-chronic Cd exposure.

In this study, we developed a “scoring system” involving the use of multiple reference genes for RT-qPCR. The main purpose of the reference gene (or so-called the housekeeping gene), is to normalize the amount of cDNA added for the RT-qPCR. A reference gene is expected to be universally expressed in all cells of an organism, and the gene expression is unaffected by the cell types, developmental stage, cell-cycle state, and most importantly, experimental treatments [25]. Although it is recommended to validate the reference gene for each experiment (e.g., by performing serum starvation and induction experiments), there is no guarantee that the validated reference gene is not affected by the experimental factors [42]. Also, many of the common housekeeping genes (e.g., *ACTB* and *GAPDH*) could potentially have abnormal expressions in cancerous cells. The problems regarding reference gene selection for RT-qPCR are addressed in Eisenberg and Levanon [43]. Here, our novel scoring system provides a less biased method to analyze gene expression data based on the 2^-ΔΔCT^ method, especially for a cohort of genes that is anticipated to have low expression fold-change value.

Tumorigenesis is promoted by genome instability with increased DNA damage and reduced DNA repair capacity [40]. Thus, based on the confirmed characteristics of the CR0 cells (transformed phenotypes, greater intrinsic DNA damage, aberrant DNA repair gene expression profile, and reduced DNA repair capacity), it is indicated that post-chronic Cd-exposed cells are highly prone to malignant transformation. The BEAS-2B cell line was regularly used to investigate the carcinogenic effects of cigarette smoke on human lung cells in many previous studies [44,45]. However, the smoking history of the individual whose primary bronchial cells were obtained to generate the cell line was not documented, and this has not been addressed in any of the previous studies. As a matter of fact, information of smoking status is usually lacking in many established cell lines, even in some of the lung cancer cell lines, which could be a disadvantage for research regarding cigarette smoke-induced carcinogenesis. Nevertheless, here we used online clinical datasets to evaluate the relationships between DNA repair genes, lung cancer, and cigarette smoking, and showed that some of the DNA repair genes might play a role in lung cancer and smoking. However, this database analysis has several limitations. First, the development of cancer is complicated and cannot be attributed to only a single (or a few) gene expression. Second, although smoking is one of the primary sources of Cd exposure in human [17,31], Cd is not the only carcinogens present in cigarettes. In fact, smokers are exposed to a toxic mixture of over 7000 chemicals, in which many are recognized as carcinogens, from cigarette smoking [46].

Studies have shown that Cd exposure may cause tumor cells to exhibit resistance to oxidants, radiation, and to the action of many chemotherapeutic agents due to up-regulation of gene and protein expression of several resistance factors, such as glutathione and metallothionein [47]. However, results obtained in our study showed that the CR0 cells were more sensitive to chemotherapeutic (cisplatin, gemcitabine, and vinorelbine tartrate) and DNA damaging (H_2_O_2_) agents than the PM control cells. These results suggest that Cd-exposed and post Cd-exposed cells are likely to respond differently toward chemotherapeutic drugs, probably due to the expression changes in the resistance factors, although further work is required to confirm this. Nevertheless, more DNA damage-related chemotherapeutic drugs and radiotherapy should be tested on the CR0 cells in order to see if the reduced DNA damage repair capacity of post Cd-exposed cells can result in a better anticancer treatment outcome. In addition, there are several other experiments that can be performed in the future to enhance our knowledge regarding the molecular mechanism of Cd-induced carcinogenicity, including studying the epigenotoxicity effects of Cd using the CR0 cell model established here. The three main epigenetic components, namely the DNA methylation, histone PTMs, and ncRNA expression, could be assessed using high-throughput sequencing or microarray techniques [48]. Recent studies have revealed that Cd exposure and smoking affect the global DNA methylation profiles in human [18,49,50,51].

Although we have shown that the CR0 cells have different transcriptomic landscapes of DNA repair and the abovementioned properties than acute/chronic Cd-exposed cells, it is less clear when the CR0 cells acquired their properties during the post-exposure period. In order to assess the dynamic changes upon post-chronic Cd exposure, future experiments could also be performed in BEAS-2B cells collected at multiple time points (e.g., 1^st^ month, 2^nd^ month, 3^rd^ month and up to 12^th^ month and more) after chronic-Cd exposure, along with their passage-matched controls.

**Figure 7:**
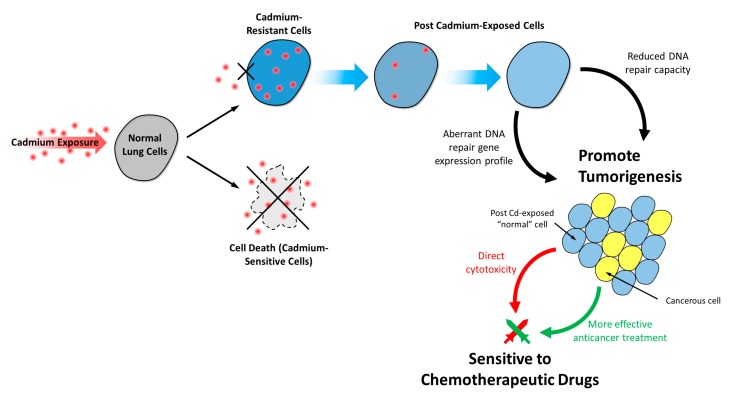
The potential long-term cytotoxic and carcinogenic effects of post-chronic Cd exposure on human lung cells. Post Cd-exposed cells have an aberrant DNA repair gene expression profile and reduced DNA repair capacity which can contribute to carcinogenesis. These post Cd-exposed cells are more sensitive to chemotherapeutic drugs, which may represent a double-edged sword: on the one hand, post Cd-exposed cells are more sensitive to chemotherapy which is beneficial in terms of anticancer treatment; on the other hand, exposure to Cd may also make the patients that have to undergo chemotherapy more vulnerable to drug toxicity.

Overall, this study unveiled a new perspective for the environmental health hazard of Cd and will encourage further research on post-Cd (and other carcinogens) exposure. Research on post-chronic Cd exposure is not only relevant to people like ex-smokers and retired workers in high-risk occupations, but also to people from the general population who live in Cd-polluted environments. In addition, our findings regarding CR0 cells’ decreased DNA repair capacity and increased sensitivity to chemotherapeutic and DNA damaging agents may have important health and clinical implications (especially for lung cancer patients). These findings suggest that Cd-induced lung cancer can be more effectively treated with chemotherapy, but this also poses a serious health concern: “normal” cells may also be more vulnerable to these chemotherapeutic drugs upon chronic or post-chronic Cd exposure. Therefore, in addition to promoting carcinogenesis, Cd exposure also has the potential long-term effects of making patients that have to undergo chemotherapy more susceptible to the toxicity of the chemotherapeutic drugs, as demonstrated in Figure 7.

## 5. Conclusions

In conclusion, this study encourages further research on the previously uncharted territory of post-Cd exposure by revealing the long-term effects of post-chronic Cd exposure on DNA repair systems in a novel post Cd-exposed cell model. We demonstrated for the first time that the human lung cell exhibits lasting high levels of DNA damage associated with Cd exposure. We showed that the post-chronic Cd-exposed human lung cells had retained Cd-resistance property, aberrant expression of DNA repair genes, reduced DNA repair capacity, and were more susceptible to chemotherapeutic and DNA damaging agents.

## Figures and Tables

**Table 1:** DNA repair genes and their associated DNA repair-related pathways investigated in this study.

Acronym	DNA Repair Pathways	No. of Genes ^1^	Gene Name (UniProtKB accession ID)
**Major DNA Repair Pathways**			
BER	Base excision repair	38	*APEX1* (P27695); *CCNO* (P22674); *ERCC1* (P07992); *ERCC4* (Q92889); *ERCC5* (P28715); *ERCC6* (Q03468); *FEN1* (P39748); *GADD45A* (P24522); *HUS1* (O60921); *LIG1* (P18858); *LIG3* (P49916); *MBD4* (O95243); *MPG* (P29372); *MUTYH* (Q9UIF7); *NEIL1* (Q96FI4); *NEIL2* (Q969S2); *NEIL3* (Q8TAT5); *NTHL1* (P78549); *OGG1* (O15527); *PARP1* (P09874); *PARP2* (Q9UGN5); *PARP3* (Q9Y6F1); *PCNA* (P12004); *POLB* (P06746); *POLD3* (Q15054); *RAD23A* (P54725); *RAD23B* (P54727); *RAD52* (P43351); *RPA1* (P27694); *RPA2* (P15927); *RPA3* (P35244); *SIRT1* (Q96EB6); *SMUG1* (Q53HV7); *TDG* (Q13569); *TP53* (P04637); *UNG* (P13051); *WRN* (Q14191); *XRCC1* (P18887)
NER	Nucleotide excision repair	37	*ATR* (Q13535); *ATXN3* (P54252); *CCNH* (P51946); *CDK7* (P50613); *CETN2* (P41208); *DDB1* (Q16531); *DDB2* (Q92466); *ERCC1* (P07992); *ERCC2* (P18074); *ERCC3* (P19447); *ERCC4* (Q92889); *ERCC5* (P28715); *ERCC6* (Q03468); *ERCC8* (Q13216); *GADD45A* (P24522); *GTF2H1* (P32780); *GTF2H2* (Q13888); *GTF2H3* (Q13889); *GTF2H4* (Q92759); *LIG1* (P18858); *LIG3* (P49916); *MMS19* (Q96T76); *NTHL1* (P78549); *OGG1* (O15527); *PCNA* (P12004); *POLD3* (Q15054); *RAD23A* (P54725); *RAD23B* (P54727); *RFC1* (P35251); *RPA1* (P27694); *RPA2* (P15927); *RPA3* (P35244); *SIRT1* (Q96EB6); *TP53* (P04637); *XAB2* (Q9HCS7); *XPA* (P23025); *XRCC1* (P18887)
MMR	Mismatch repair	19	*ABL1* (P00519); *EXO1* (Q9UQ84); *MLH1* (P40692); *MLH3* (Q9UHC1); *MSH2* (P43246); *MSH3* (P20585); *MSH4* (O15457); *MSH5* (O43196); *MSH6* (P52701); *PCNA* (P12004); *PMS1* (P54277); *PMS2* (P54278); *POLD3* (Q15054); *RFC1* (P35251); *RPA1* (P27694); *RPA2* (P15927); *RPA3* (P35244); *TDG* (Q13569); *TREX1* (Q9NSU2)
HRR	Homologous recombinational repair	49	*ATM* (Q13315); *ATR* (Q13535); *BLM* (P54132); *BRCA1* (P38398); *BRCA2* (P51587); *BRIP1* (Q9BX63); *CHEK1* (O14757); *DMC1* (Q14565); *ERCC1* (P07992); *ERCC4* (Q92889); *ERCC6* (Q03468); *FANCA* (O15360); *FANCB* (Q8NB91); *FANCF* (Q9NPI8); *FANCG* (O15287); *FEN1* (P39748); *GADD45A* (P24522); *H2AFX* (P16104); *HUS1* (O60921); *LIG3* (P49916); *MDC1* (Q14676); *MRE11* (P49959); *MSH2* (P43246); *MSH4* (O15457); *MSH5* (O43196); *MSH6* (P52701); *NBN* (O60934); *PARP1* (P09874); *PARP2* (Q9UGN5); *PARP3* (Q9Y6F1); *RAD17* (O75943); *RAD21* (O60216); *RAD50* (Q92878); *RAD51* (Q06609); *RAD51B* (O15315); *RAD51C* (O43502); *RAD51D* (O75771); *RAD52* (P43351); *RAD54B* (Q9Y620); *RAD54L* (Q92698); *REV1* (Q9UBZ9); *RPA1* (P27694); *RPA2* (P15927); *RPA3* (P35244); *SIRT1* (Q96EB6); *TP53BP1* (Q12888); *WRN* (Q14191); *XRCC1* (P18887); *XRCC2* (O43543)
NHEJ	Non-homologous end-joining	22	*ATM* (Q13315); *ATP23* (Q9Y6H3); *BRCA1* (P38398); *ERCC1* (P07992); *ERCC4* (Q92889); *LIG4* (P49917); *MDC1* (Q14676); *MLH1* (P40692); *MRE11* (P49959); *NBN* (O60934); *PARP1* (P09874); *PARP2* (Q9UGN5); *PARP3* (Q9Y6F1); *PRKDC* (P78527); *RAD50* (Q92878); *SIRT1* (Q96EB6); *TP53BP1* (Q12888); *WRN* (Q14191); *XRCC1* (P18887); *XRCC4* (Q13426); *XRCC5* (P13010); *XRCC6* (P12956)
**Other DNA Repair Pathways**			
POL	Polymerases	4	*PCNA* (P12004); *POLB* (P06746); *POLD3* (Q15054); *REV1* (Q9UBZ9)
DRD	Direct reversal of damage	3	*ALKBH1* (Q13686); *ALKBH3* (Q96Q83); *MGMT* (P16455)
SMNP	Sanitization/modulation of nucleotide pools	3	*DUT* (P33316); *NUDT1* (P36639); *RRM2B* (Q7LG56)
FA	Fanconi anemia	9	*BRCA1* (P38398); *BRCA2* (P51587); *BRIP1* (Q9BX63); *FANCA* (O15360); *FANCB* (Q8NB91); *FANCF* (Q9NPI8); *FANCG* (O15287); *RAD51* (Q06609); *RAD51C* (O43502)
TLM	Telomere maintenance	26	*ATR* (Q13535); *BRCA1* (P38398); *BRCA2* (P51587); *ERCC1* (P07992); *ERCC4* (Q92889); *FEN1* (P39748); *MRE11* (P49959); *NBN* (O60934); *PARP1* (P09874); *PARP3* (Q9Y6F1); *PCNA* (P12004); *POLD3* (Q15054); *RAD50* (Q92878); *RAD51* (Q06609); *RAD51C* (O43502); *RAD51D* (O75771); *RFC1* (P35251) *RPA1* (P27694); *RPA2* (P15927); *RPA3* (P35244); *SIRT1* (Q96EB6); *TP53* (P04637); *WRN* (Q14191); *XRCC1* (P18887); *XRCC5* (P13010); *XRCC6* (P12956)
TLS	Translesion synthesis	3	*PCNA* (P12004); *POLD3* (Q15054); *REV1* (Q9UBZ9)
CSM	Chromatin structure and modification	16	*CHEK1* (O14757); *ERCC6* (Q03468); *FEN1* (P39748); *H2AFX* (P16104); *LIG3* (P49916); *PCNA* (P12004); *RAD17* (O75943); *RAD21* (O60216); *RAD51* (Q06609); *RAD51C* (O43502); *RPA1* (P27694); *RPA2* (P15927); *RPA3* (P35244); *SIRT1* (Q96EB6); *TP53* (P04637); *XRCC1* (P18887)
MMEJR	Microhomology-mediated end-joining repair	6	*FEN1* (P39748); *LIG3* (P49916); *MRE11* (P49959); *NBN* (O60934); *PARP1* (P09874); *XRCC1* (P18887)
SDA	Genes defective in diseases associated with sensitivity to DNA-damaging agents	7	*ATM* (Q13315); *ATXN3* (P54252); *BLM* (P54132); *ERCC6* (Q03468); *ERCC8* (Q13216); *NBN* (O60934); *WRN* (Q14191)
UBM	Ubiquitination and modification (Rad6 pathways)	6	*RAD18* (Q9NS91); *SHPRH* (Q149N8); *UBE2A* (P49459); *UBE2B* (P63146); *UBE2N* (P61088); *UBE2V2* (Q15819)
OTHER	Other DNA damage-related	4	*CRY1* (Q16526); *SUMO1* (P63165); *TOP3A* (Q13472); *TOP3B* (O95985)

^1^ These numbers add up to over 117 (the total number of genes studied) because some of the genes are involved in more than one pathway.

## Supplementary Materials

The following are available online at https://www.mdpi.com/2073-4409/8/8/842/s1. **Table S1**: Information on oligonucleotide primers used in this study. **Table S2**: Gene expression of 117 DNA repair-related genes individually normalized to six different reference genes in CR0 over PM cells. **Table S3**: Gene expression of 12 selected DNA repair genes individually normalized to six different reference genes in PM and CR0 cells upon exposure to 20 µM of CdCl_2_ for 72 h. **Table S4**: Expression of 12 differentially expressed DNA repair genes in lung cancer patients obtained from the online database and grouped by patients’ smoking status. **Figure S1**: Determination of the Cd-resistance of the post-chronic Cd-exposed cells. (**A**) Morphology foci of PM and CR0 cells treated with 20 µM of CdCl_2_ and grown for seven days were observed using a light microscope and photographed. (**B**) Cell viability of PM, CR0, and Cd-resistance BEAS-2B cells (maintained in 20 µM CdCl_2_) exposed to different concentrations of CdCl_2_ for 72 h was measured by MTS assay (absorbance at 495 nm). **Figure S2**: Western blot of histones extracted from CR0 and PM cells probed with H2AFX (1:1000, A11361, ABClonal), γH2AFX (1:1000, AP0099, ABClonal), or H3 (1:2000, 4499, CST) antibody. Marker used was PageRuler™ Prestained Protein Ladder (Thermo Scientific, USA). **Figure S3**: Volcano plots of 117 DNA repair gene expression profile of CR0 over PM cells when individually normalized to six different references genes: (**A**) *ACTB*, (**B**) *B2M*, (**C**) *GAPDH*, (**D**) *HPRT1*, (**E**) *PRLP0*, and (**F**) 5HKGs (based on the average of *ACTB*, *B2M*, *GAPDH*, *HPRT1*, and *PRLP0*). Significantly differentially expressed genes (*p* ≤ 0.05 and fold-change ≥1.5) are highlighted in green (down-regulated) or red (up-regulated). **Figure S4**: Differentially expressed DNA repair-related genes of the CR0 cells over control PM cells. (**A**) Venn-diagram shows the number of genes that were significantly up- or down-regulated (*p* ≤ 0.05 and fold-change ≥1.5 when normalized to either one of the six reference genes; numbers in overlapping set ≥4 (*s*-score ≥ 4) are highlighted in red and asterisk. (**B**) Number of genes with significantly up- or down-regulated expression in CR0 cells. (**C**) The 12 differentially expressed DNA repair genes and their associated DNA repair pathways. **Figure S5**: Protein expressions of selected DNA repair genes were determined by Western immunoblotting. Antibodies were purchased from Santa Cruz Biotechnology: ATR (sc-515173); ATM (sc-377293); CDK7 (sc-365075); ERCC1 (sc-17809); LIG3 (sc-390922); NEIL1 (sc-271164); NEIL3 (sc-393703); PARP1 (sc-8007); PMS2 (sc-25315); SMUG1 (sc-377370); SUMO1 (sc-5308), GeneTex: ERCC2 (GTX108948); TP53 (GTX70214), Sigma-Aldrich: ACTB (A5441), ABClonal: H2AFX (A11361), and Cell Signaling Technology: H3 (4499). The H3 was used as a control for H2AFX whereas ACTB was used for the rest of the proteins. Experiments were carried out using different batches of samples and thus the multiple ACTB data. **Figure S6**: Expression of the 12 differentially expressed DNA repair genes in lung cancer patients obtained from TCGA datasets and visualized using the cBioPortal database. (**A**) Data from 517 lung adenocarcinoma samples (TCGA, provisional). (**B**) Data from 501 lung squamous cell carcinoma samples (TCGA, provisional). Each bar represents an individual patient: bars highlighted in red represent up-regulated gene expression (z-score >+1.5) whereas those highlighted in blue represent down-regulated gene expression (z-score <−1.5); grey bars represent no significant differences in gene expression between the diseased and healthy control samples (z-score is between −1.5 and +1.5). For both datasets, the mRNA expression z-score threshold is set at ±1.5.
