# Supplementary material for: Lasting DNA Damage and Aberrant DNA Repair Gene Expression Profile Are Associated with Post-Chronic Cadmium Exposure in Human Bronchial Epithelial Cells
Source: Cells. 2019 Aug 6;8(8):842. doi: 10.3390/cells8080842 (PMC6721754; doi:10.3390/cells8080842)
Supplement: Supplementary file 1 [file cells-08-00842-s001.zip › cells-548050-SI.pdf]

Supporting

Table S1. Information on oligonucleotide primers used in this study.

Gene	Primer
Reference (housekeeping) genes	
**ACTB**	RT^2 Profiler™ PCR Arrays (Human DNA Repair)
	F: CTGGAACGGTGAAGGTGACA
	R: AAGGGACTTCCTGTAACAATGCA
**B2M**	RT^2 Profiler™ PCR Arrays (Human DNA Repair)
	F: CCTGGAGGCTATCCAGCGTA
	R: CGGATGGATGAAACCCAGACA
**GAPDH**	RT^2 Profiler™ PCR Arrays (Human DNA Repair)
	F: AACAGCGACACCCACTCCTC
	R: CATACCAGGAAATGAGCTTGACAA
**HPRT1**	RT^2 Profiler™ PCR Arrays (Human DNA Repair)
	F: TGACACTGGCAAAACAATGCA
	R: GGTCCTTTTCACCAGCAAGCT
**PRLP0**	RT^2 Profiler™ PCR Arrays (Human DNA Repair)
	F: ACCTCCTTTTTCCAGGCTTT
	R: CCCACTTTGTCTCCAGTCTTG
DNA repair-related genes	
**ABL1**	F: TTCAGCGGCCAGTAGCATCTGACTT
	R: CTGTTGACTGGCGTGATGTAGTTGCTT
**ALKBH1**	F: ACTTGAAGACCGCTCGTGTT
	R: TCCTGGCCCGTTTTACTTCG
**ALKBH3**	F: GGAGGTGCCCCATTATTGCT
	R: AGGTCAGGTTCACTCTCGGT
**APEX1**	RT^2 Profiler™ PCR Arrays (Human DNA Repair)
**APEX2**	RT^2 Profiler™ PCR Arrays (Human DNA Repair)*
**ATM**	RT^2 Profiler™ PCR Arrays (Human DNA Repair)

**ATP23**	RT^2 Profiler™ PCR Arrays (Human DNA Repair)
**ATR**	RT^2 Profiler™ PCR Arrays (Human DNA Repair)
**ATXN3**	RT^2 Profiler™ PCR Arrays (Human DNA Repair)
**BLM**	F: AGCACTGCTGTGAAAGATCAGA
	R: TGGGTTGATAGGCAGCTGTG
**BRCA1**	RT^2 Profiler™ PCR Arrays (Human DNA Repair)
**BRCA2**	RT^2 Profiler™ PCR Arrays (Human DNA Repair)
**BRIP1**	RT^2 Profiler™ PCR Arrays (Human DNA Repair)
**CCNH**	RT^2 Profiler™ PCR Arrays (Human DNA Repair)
**CCNO**	RT^2 Profiler™ PCR Arrays (Human DNA Repair)
**CDK7**	RT^2 Profiler™ PCR Arrays (Human DNA Repair)
**CETN2**	F: GGCAAACATGGCATCAAGTTCT
	R: GCTCTTCAGTAAGCTCAGGCT
**CHEK1**	F: TTGGTTGACTTCCGGCTTTCT
	R: GCCAAATCTTCTGGCTGCTC
**CRY1**	F: CAGGTGGCGATTTTTGCTTCA
	R: TCCAGCTTCAGTTGCCAGTT
**DDB1**	RT^2 Profiler™ PCR Arrays (Human DNA Repair)
**DDB2**	RT^2 Profiler™ PCR Arrays (Human DNA Repair)
**DMC1**	RT^2 Profiler™ PCR Arrays (Human DNA Repair)
**DUT**	F: GAAGAGTGGCTCCACGGTCA
	R: AAATCCGTTCGCAAATGAGCTG
**ERCC1**	RT^2 Profiler™ PCR Arrays (Human DNA Repair)
**ERCC2**	RT^2 Profiler™ PCR Arrays (Human DNA Repair)
**ERCC3**	RT^2 Profiler™ PCR Arrays (Human DNA Repair)
**ERCC4**	RT^2 Profiler™ PCR Arrays (Human DNA Repair)

**ERCC5**	RT2 Profiler™ PCR Arrays (Human DNA Repair)	
**ERCC6**	RT2 Profiler™ PCR Arrays (Human DNA Repair)	
**ERCC8**	RT2 Profiler™ PCR Arrays (Human DNA Repair)	
**EXO1**	RT2 Profiler™ PCR Arrays (Human DNA Repair)	
**FANCA**	F: CGCAGGTCACGGTTGATGTA	
	R: TGAACACTCCGAACCAGCAC	
**FANCB**	F: GCAGAGGTGTGAAGTGAGCA	
	R: AGGGCACCACTCACTTTTAGG	
**FANCF**	F: CCTGGGAGGAGTTGCACAAT	
	R: TGCGCTTTACAGGTCTCCAG	
**FANCG**	F: TAGTTGAGGCCTTGAATGTC	
	R: CTTGCTAGTATGTGCTTGGT	
**FEN1**	RT2 Profiler™ PCR Arrays (Human DNA Repair)	
**GADD45A**	F: GAGAGCAGAAGACCGAAAGGA	
	R: CACAACACCACGTTATCGGG	
**GTF2H1**	F: AAAACGTGAGGTGGCCATGA	
	R: CAGGCCAGGTTTCCAGAGTT	
**GTF2H2**	F: TCCTCAGCACACCATTGCTT	
	R: GCTCACAGTACTTTGCCCGA	
**GTF2H3**	F: CCACCCCCAGTTCATGTTGA	
	R: AAAGGCTGTCTCGCACGTAG	
**GTF2H4**	F: GCTGCAGATTGCCCTCATTG	
	R: GGAGTCTGTCCCTTTCCAGC	
**H2AFX**	F: AGAAGACGCGAATCATCCCC	
	R: CGGGCCCTCTTAGTACTCCT	
**HUS1**	F: AACCACTTCACACGAATCAGTAAC	
	R: GCAGAGACACCCTCCATTTGA	
**LIG1**	RT2 Profiler™ PCR Arrays (Human DNA Repair)	
**LIG3**	RT2 Profiler™ PCR Arrays (Human DNA Repair)	
**LIG4**	RT2 Profiler™ PCR Arrays (Human DNA Repair)	
**MBD4**	F: AGCTGAGGTAGCAAGAACCG	
	R: ATTCGGTAAGAGTCGTTGCCA	
**MDC1**	F: GCTGACTTGCTCTGCCAGTA	

	R: CTTCCTCCTCCGAGTCCTCA
**MGMT**	RT^2 Profiler™ PCR Arrays (Human DNA Repair)
**MLH1**	RT^2 Profiler™ PCR Arrays (Human DNA Repair)
**MLH3**	RT^2 Profiler™ PCR Arrays (Human DNA Repair)
**MMS19**	RT^2 Profiler™ PCR Arrays (Human DNA Repair)
**MPG**	RT^2 Profiler™ PCR Arrays (Human DNA Repair)
**MRE11A**	RT^2 Profiler™ PCR Arrays (Human DNA Repair)
**MSH2**	RT^2 Profiler™ PCR Arrays (Human DNA Repair)
**MSH3**	RT^2 Profiler™ PCR Arrays (Human DNA Repair)
**MSH4**	RT^2 Profiler™ PCR Arrays (Human DNA Repair)
**MSH5**	RT^2 Profiler™ PCR Arrays (Human DNA Repair)
**MSH6**	RT^2 Profiler™ PCR Arrays (Human DNA Repair)
**MUTYH**	RT^2 Profiler™ PCR Arrays (Human DNA Repair)
**NBN**	F: GAGCCTTTGGTTGCATGCTC
	R: GAGGCTGCTTCTTGGACTCA
**NEIL1**	RT^2 Profiler™ PCR Arrays (Human DNA Repair)
**NEIL2**	RT^2 Profiler™ PCR Arrays (Human DNA Repair)
**NEIL3**	RT^2 Profiler™ PCR Arrays (Human DNA Repair)
**NTHL1**	RT^2 Profiler™ PCR Arrays (Human DNA Repair)
**NUDT1**	F: CCGACGACAGCTACTGGTTT
	R: AGTCCAGGATGGTGTCCTGA
**OGG1**	RT^2 Profiler™ PCR Arrays (Human DNA Repair)
**PARP1**	RT^2 Profiler™ PCR Arrays (Human DNA Repair)

**PARP2**	RT^2 Profiler™ PCR Arrays (Human DNA Repair)
**PARP3**	RT^2 Profiler™ PCR Arrays (Human DNA Repair)
**PCNA**	F: GTAGTAAAGATGCCTTCTGGTG
	R: TCTCTATGGTAACAGCTTCCTC
**PMS1**	RT^2 Profiler™ PCR Arrays (Human DNA Repair)
**PMS2**	RT^2 Profiler™ PCR Arrays (Human DNA Repair)
**PNKP**	RT^2 Profiler™ PCR Arrays (Human DNA Repair)
**POLB**	RT^2 Profiler™ PCR Arrays (Human DNA Repair)
**POLD3**	RT^2 Profiler™ PCR Arrays (Human DNA Repair)
**POLL**	RT^2 Profiler™ PCR Arrays (Human DNA Repair)
**PRKDC**	RT^2 Profiler™ PCR Arrays (Human DNA Repair)
**RAD17**	F: ATCAGGTAACAGACTGGGTTGAC
	R: TCACACCTAATGATGTGGCAGT
**RAD18**	RT^2 Profiler™ PCR Arrays (Human DNA Repair)
**RAD21**	RT^2 Profiler™ PCR Arrays (Human DNA Repair)
**RAD23A**	RT^2 Profiler™ PCR Arrays (Human DNA Repair)
**RAD23B**	RT^2 Profiler™ PCR Arrays (Human DNA Repair)
**RAD50**	RT^2 Profiler™ PCR Arrays (Human DNA Repair)
**RAD51**	RT^2 Profiler™ PCR Arrays (Human DNA Repair)
**RAD51B**	RT^2 Profiler™ PCR Arrays (Human DNA Repair)
**RAD51C**	RT^2 Profiler™ PCR Arrays (Human DNA Repair)
**RAD51D**	RT^2 Profiler™ PCR Arrays (Human DNA Repair)
**RAD52**	RT^2 Profiler™ PCR Arrays (Human DNA Repair)

**RAD54B**	F: CAGCACCAAGTCAGTTGCAG
	R: TTGTGACGGGAGAAAGGTGTT
**RAD54L**	RT^2 Profiler™ PCR Arrays (Human DNA Repair)
**REV1**	F: GAGACCTCCAGCACCCAATC
	R: AACCGATTCCACCGATTGCT
**RFC1**	RT^2 Profiler™ PCR Arrays (Human DNA Repair)
**RPA1**	RT^2 Profiler™ PCR Arrays (Human DNA Repair)
**RPA2**	F: GCAGGCCACCTGAGATCTTT
	R: ATAGGTGCTCTCCCTGCTGA
**RPA3**	RT^2 Profiler™ PCR Arrays (Human DNA Repair)
**RRM2B**	F: TGGTGGAGCGCTTTAGTCAG
	R: CTATCCATCGCAAGGCCCAA
**SHPRH**	F: AGTACCCAAATTCTGGGCCG
	R: TCCCTCGGGAAGAGTGAGAG
**SIRT1**	F: ACAGGTTGCGGGAATCCAAA
	R: GTTCATCAGCTGGGCACCTA
**SLK**	RT^2 Profiler™ PCR Arrays (Human DNA Repair)
**SMUG1**	RT^2 Profiler™ PCR Arrays (Human DNA Repair)
**SUMO1**	F: GGTCTGGACCAAAAGAAGAGGAA
	R: AACCCATATCCCAGGCCAAAA
**TDG**	RT^2 Profiler™ PCR Arrays (Human DNA Repair)
**TOP3A**	RT^2 Profiler™ PCR Arrays (Human DNA Repair)
**TOP3B**	RT^2 Profiler™ PCR Arrays (Human DNA Repair)
**TP53**	F: TAACAGTTCCTGCATGGGCG
	R: ACCTCAAAGCTGTTCCGTCC
**TP53BP1**	F: CATGGGAGTTCTCTCAGGCAAA
	R: CTCCTGCCCCTACAGGTTTTAC
**TREX1**	RT^2 Profiler™ PCR Arrays (Human DNA Repair)
**UBE2A**	F: ACCCAATCCCAATAGTCCAGC
	R: TCACGCCAGCTTTGTTCTACT
**UBE2B**	F: CGGTTACAAGAGGACCCACC

	R: GGTGTCCCTTCTGGTCCAAAT
**UBE2N**	F: AGCCCAGACATCTTCAGTCC<br>R: TAAACCAGGATGGGGGAAAT
**UBE2V2**	F: GGCATGATTATTGGGCCACC<br>R: TGCTAACACTGGTATGCTCCG
**UNG**	RT^2 Profiler™ PCR Arrays (Human DNA Repair)
**WRN**	F: GCATGCACTTATCCCAAGCG<br>R: GTTGAAGTCCGCTGTCAGGA
**XAB2**	RT^2 Profiler™ PCR Arrays (Human DNA Repair)
**XPA**	RT^2 Profiler™ PCR Arrays (Human DNA Repair)
**XPC**	RT^2 Profiler™ PCR Arrays (Human DNA Repair)*
**XRCC1**	RT^2 Profiler™ PCR Arrays (Human DNA Repair)
**XRCC2**	RT^2 Profiler™ PCR Arrays (Human DNA Repair)
**XRCC3**	RT^2 Profiler™ PCR Arrays (Human DNA Repair)*
**XRCC4**	RT^2 Profiler™ PCR Arrays (Human DNA Repair)
**XRCC5**	RT^2 Profiler™ PCR Arrays (Human DNA Repair)
**XRCC6**	RT^2 Profiler™ PCR Arrays (Human DNA Repair)

*Genes are excluded due to inefficient PCR amplification, bad melt curve, or insufficient replicated samples.

Note 1: RT2 Profiler™ PCR Arrays (Human DNA Repair) kit was purchased from Qiagen-SABiosciences.

Note 2: optimal Tm for all the above primer sets is 60°C; the amplicon length and GC content for each primer are ~50–300bp and 35–65%, respectively.

Table S2. Gene expression of 117 DNA repair-related genes individually normalized to six different reference genes in CR0 over PM cells.

**Genes**	**ACTB**		**B2M**		**GAPDH**		**HPRT1**		**RPLP0**		**5HKGs**[a]		***S*-score**[c]
	**FC**[b]	***P*-value**	**FC**	***P*-value**	**FC**	***P*-value**	**FC**	***P*-value**	**FC**	***P*-value**	**FC**	***P*-value**	
**ABL1**[d]	**-2.4325**	***0.0008***	**-2.5046**	***0.0016***	**-2.0376**	***0.0054***	**-3.1673**	***0.0009***	**-3.0516**	***0.0004***	**-2.6062**	***0.0004***	**6**

ALKBH1	1.0808	0.1735	1.0624	0.4413	1.3264	0.0736	-1.2545	0.0556	-1.1881	0.1234	1.0045	0.9336	0
ALKBH3	1.447	**0.0183**	1.4082	**0.0006**	**1.795**	**0.0354**	1.0576	0.5722	1.1206	0.4441	1.3415	**0.0373**	1
APEX1	-1.2581	0.6011	1.0872	0.8097	1.3555	0.471	1.1604	0.6882	1.2256	0.5333	1.0896	0.814	0
ATM	**-1.5752**	0.1807	-1.139	0.2897	1.0746	0.8303	-1.0694	0.6436	1.0077	0.9536	-1.1376	0.5382	0
ATP23	**-2.0604**	**0.0107**	-1.4117	**0.0409**	-1.181	0.4861	-1.34	**0.0225**	-1.2206	0.0859	-1.4356	**0.0229**	1
ATR	-1.4854	0.1863	-1.0618	0.7054	1.1581	0.593	1.0003	0.9984	1.0725	0.5546	-1.0656	0.7249	0
ATXN3	**-1.704**	**0.0239**	-1.1555	0.4627	1.024	0.9141	-1.0978	0.5677	1.0038	0.9805	-1.18	0.2786	1
BLM	1.014	0.9326	1.0044	0.9793	1.2221	0.2051	-1.3129	0.2465	-1.2693	0.3072	-1.0606	0.7312	0
BRCA1	**-2.199**	**0.0216**	**-1.5173**	**0.0433**	-1.2325	0.3164	-1.4386	0.0609	-1.3249	0.1176	**-1.5345**	**0.0343**	3
BRCA2	**-1.6708**	0.1298	-1.1021	0.6447	1.0965	0.6497	-1.0497	0.8068	1.036	0.8575	-1.1314	0.5212	0
BRIP1	**-1.622**	0.1241	-1.1477	0.3339	1.0782	0.7707	-1.0852	0.5925	-1.0088	0.9462	-1.1538	0.4179	0
CCNH	**-1.5556**	0.1775	-1.0585	0.7086	1.0717	0.8278	-1.0118	0.9384	1.0658	0.6588	-1.0957	0.6445	0
CCNO	**-1.7519**	0.1528	-1.1899	0.5648	-1.0533	0.8926	-1.1442	0.6115	-1.032	0.9059	-1.2302	0.4896	0
CDK7	-1.3797	0.2074	1.0505	0.527	1.2571	0.3721	1.1067	0.1512	1.201	**0.0123**	1.0325	0.7744	0
CETN2	1.1719	0.0894	1.1435	0.0562	1.4491	0.0517	-1.1614	0.2158	-1.0979	0.4718	1.0881	0.3086	0
CHEK1	1.1413	0.2103	1.1135	0.1069	1.4055	0.1019	-1.192	0.1402	-1.1305	0.4135	1.0582	0.5739	0
CRY1	-1.3417	**0.0411**	-1.3685	0.1001	-1.1209	0.4907	**-1.7897**	**0.0081**	**-1.7175**	**0.0127**	-1.447	**0.0306**	2
DDB1	-1.2656	0.4987	1.2871	0.4626	1.4566	0.1258	1.3208	0.3817	1.423	0.2659	1.2062	0.4962	0
DDB2	-1.2026	0.6081	1.2276	0.4318	1.4292	0.3182	1.2967	0.3699	1.3675	0.2136	1.19	0.5283	0
DMC1	**-1.5425**	0.2054	-1.0048	0.9805	1.1583	0.6666	1.0254	0.8798	1.1327	0.5802	-1.0461	0.8368	0
**DUT**	**1.6653**	**0.0004**	**1.6142**	**0.0008**	**2.0473**	**0.01**	1.2192	**0.0049**	1.3	**0.0075**	**1.5428**	**0.0009**	**4**
ERCC1	**-1.5645**	0.3325	1.3203	0.6535	1.2749	0.5471	1.2917	0.6415	1.4379	0.5351	1.1318	0.7982	0

ERCC2	**-1.8267**	0.444	1.2268	0.7085	1.1268	0.8067	1.258	0.6798	1.3187	0.633	1.0349	0.9506	0
ERCC3	-1.2267	0.5207	1.2866	0.4879	**1.5068**	0.1659	1.3394	0.3554	**1.5**	0.2173	1.2371	0.4507	0
ERCC4	**-1.968**	**0.0278**	-1.2215	0.5025	-1.0865	0.6344	-1.1792	0.5592	-1.0827	0.7702	-1.2942	0.2817	1
ERCC5	**-1.9898**	0.0562	-1.3938	0.0945	-1.1307	0.6103	-1.3122	0.1352	-1.2167	0.1924	-1.4024	0.103	0
ERCC6	**-1.6267**	0.1267	-1.227	0.171	1.0435	0.8926	-1.1434	0.4385	-1.0644	0.6508	-1.2009	0.3807	0
ERCC8	-1.3036	0.384	1.0568	0.7362	1.3351	0.377	1.1209	0.4509	1.228	0.2095	1.066	0.7466	0
EXO1	**-1.8734**	**0.0424**	-1.3271	0.0724	-1.0774	0.7539	-1.2562	0.116	-1.1635	0.191	-1.3349	0.0903	1
FANCA	-1.2024	0.2986	-1.2235	0.2775	-1.0052	0.9732	**-1.5781**	0.0768	**-1.5268**	0.079	-1.29	0.1708	0
FANCB	1.0511	0.6895	1.0294	0.7162	1.2926	0.208	-1.2919	0.0985	-1.229	0.2642	-1.0255	0.8378	0
FANCF	**1.5312**	**0.0009**	1.4943	**0.0022**	**1.8718**	**0.0039**	1.1334	0.3436	1.195	0.1372	1.4215	**0.0026**	2
FANCG	-1.1997	0.3967	-1.2352	0.3522	-1.0009	0.9964	**-1.5673**	0.1082	**-1.5132**	0.1056	-1.2867	0.2505	0
FEN1	**-1.573**	0.067	-1.0635	0.6507	1.1139	0.5834	-1.0138	0.89	1.071	0.4012	-1.091	0.3895	0
**GADD45A**	**2.6134**	**0.012**	**2.5945**	**0.0302**	**3.1287**	**0.0023**	**1.9593**	0.0812	**2.0409**	**0.0345**	**2.4294**	**0.0168**	**5**
GTF2H1	1.3366	**0.0178**	1.2991	0.0577	**1.6283**	**0.0474**	-1.0202	0.7163	1.0405	0.6693	1.2361	0.0675	1
**GTF2H2**	**2.0332**	**0**	**1.9943**	**0.0002**	**2.4756**	**0.0008**	**1.5019**	**0.0056**	**1.5806**	**0.0011**	**1.8855**	**0**	**6**
GTF2H3	-1.3859	**0.038**	-1.4202	**0.0025**	-1.1276	0.5294	**-1.8684**	**0.0049**	**-1.7796**	**0.0199**	-1.4913	**0.0176**	2
GTF2H4	**1.8208**	**0.0505**	**1.8054**	0.1015	**2.1809**	**0.0105**	1.3634	0.3192	1.4259	0.1806	**1.6932**	0.075	2
**H2AFX**	**2.0317**	**0.0142**	**1.9573**	**0.0412**	**2.4897**	**0.0001**	**1.5176**	0.2164	**1.6184**	0.0714	**1.8932**	**0.0235**	**4**
HUS1	-1.1304	0.1422	-1.156	**0.0203**	1.0831	0.6119	**-1.5268**	**0.0064**	-1.4509	**0.0273**	-1.2172	**0.0415**	1
LIG1	**-3.23**	**0.0051**	**-1.8528**	**0.0458**	**-1.8159**	0.1095	**-1.7736**	0.0637	**-1.6359**	0.1109	**-2.0253**	**0.0249**	3
**LIG3**	**1.7251**	0.2417	**2.489**	**0.0278**	**3.2031**	**0.0136**	**2.6081**	**0.0201**	**2.8339**	**0.0116**	**2.4743**	**0.0214**	**5**
LIG4	**-1.8176**	0.073	-1.2804	**0.0305**	-1.0621	0.835	-1.2118	0.1763	-1.1339	0.2833	-1.2964	0.169	0
**MBD4**	**1.7097**	**0.0024**	**1.673**	**0.0002**	**2.0825**	**0.0104**	1.2624	0.0696	1.3257	0.076	**1.584**	**0.0045**	**4**
MDC1	-1.1189	0.272	-1.1296	0.3502	1.074	0.6173	-1.4971	**0.0201**	-1.4349	**0.0332**	-1.2042	0.1099	0
MGMT	**-1.5879**	**0.0387**	-1.0634	0.7643	1.0995	0.6041	-1.0103	0.9505	1.0831	0.5722	-1.0931	0.5098	1

MLH1	**-1.5478**	*0.0704*	-1.0346	*0.8696*	1.1152	*0.5934*	1.0122	*0.9396*	1.0991	*0.4733*	-1.0705	*0.6304*	0
MLH3	**-1.5348**	*0.1646*	-1.145	*0.4832*	1.1056	*0.7375*	-1.0713	*0.7311*	1.0043	*0.9775*	-1.1284	*0.5715*	0
MMS19	**-2.2842**	***0.004***	-1.4703	***0.041***	-1.3304	*0.2325*	-1.4039	***0.05***	-1.3014	*0.0799*	**-1.5471**	***0.0125***	2
MPG	**-1.8046**	***0.0074***	-1.1694	*0.4292*	-1.0404	*0.8234*	-1.1177	*0.4635*	-1.0232	*0.8574*	-1.2227	*0.1106*	1
MRE11A	**-1.6168**	*0.1005*	-1.1615	*0.2243*	1.0468	*0.8776*	-1.0959	*0.4345*	-1.0097	*0.9223*	-1.1647	*0.3737*	0
MSH2	**-1.6022**	*0.0794*	-1.1072	*0.4181*	1.0681	*0.7868*	-1.0488	*0.7201*	1.026	*0.7979*	-1.1295	*0.3937*	0
MSH3	-1.4759	*0.1956*	-1.0725	*0.5327*	1.1493	*0.6482*	-1.0041	*0.9759*	1.0713	*0.478*	-1.0682	*0.7122*	0
MSH4	**1.5966**	*0.3866*	**2.0562**	*0.0933*	**2.9024**	*0.0847*	**2.2594**	*0.0874*	**2.4257**	*0.0599*	**2.1731**	*0.1083*	0
MSH5	-1.0763	*0.8488*	**1.7534**	*0.289*	**1.6767**	*0.2493*	**1.7806**	*0.2446*	**2.0053**	*0.1783*	**1.5533**	*0.31*	0
MSH6	-1.3769	*0.2716*	1.0495	*0.5961*	1.2629	*0.384*	1.1089	*0.3412*	1.1925	***0.0268***	1.0329	*0.8195*	0
MUTYH	**-2.082**	***0.0362***	-1.4255	*0.076*	-1.1903	*0.4591*	-1.3551	*0.1315*	-1.2709	*0.1655*	-1.4583	*0.0728*	1
NBN	**-2.3529**	***0.0138***	-1.0082	*0.9256*	1.2544	*0.1266*	-1.3275	***0.0246***	-1.2533	***0.0323***	-1.0553	*0.3105*	1
**NEIL1**	**-3.3879**	***0.0069***	**-2.239**	***0.0228***	**-1.9972**	*0.0946*	**-2.2035**	***0.0214***	**-2.0058**	***0.042***	**-2.3577**	***0.0177***	**5**
NEIL2	-1.4366	*0.2144*	1.2844	*0.5492*	1.2729	*0.4969*	1.3309	*0.5074*	1.4523	*0.409*	1.1535	*0.6778*	0
NEIL3	-1.1078	*0.688*	1.337	***0.0351***	**1.5492**	*0.1299*	1.3999	***0.0027***	**1.5187**	***0.0004***	1.2965	*0.0593*	1
NTHL1	**-1.5475**	*0.1404*	-1.0197	*0.9332*	1.0875	*0.7583*	1.0339	*0.8721*	1.1236	*0.5249*	-1.0629	*0.7625*	0
NUDT1	1.3775	*0.1999*	1.3599	*0.3091*	**1.6481**	***0.0174***	1.0403	*0.8944*	1.0879	*0.7169*	1.2839	*0.3062*	1
OGG1	-1.1361	*0.5393*	1.3154	*0.164*	**1.5337**	*0.0943*	1.3838	*0.0658*	**1.5211**	***0.0299***	1.28	*0.0882*	1
PARP1	**-3.3445**	***0.0061***	-1.4047	*0.3232*	-1.3948	*0.0928*	-1.3828	*0.2643*	-1.248	*0.4301*	**-1.5644**	*0.0765*	1
PARP2	**-1.5885**	*0.1529*	-1.1041	*0.3943*	1.1009	*0.7116*	-1.0438	*0.7495*	1.026	*0.8046*	-1.1188	*0.5017*	0
PARP3	-1.1849	*0.6302*	1.3812	*0.325*	1.4289	*0.3018*	1.4187	*0.2602*	**1.5626**	*0.1808*	1.2774	*0.3938*	0
**PCNA**	**2.0504**	***0.0118***	**1.9703**	***0.0029***	**2.5515**	***0.0253***	1.4889	***0.0263***	**1.5913**	***0.047***	**1.8963**	***0.0153***	**5**
PMS1	**-2.0039**	*0.0596*	-1.3967	***0.0078***	-1.161	*0.6172*	-1.3222	***0.0474***	-1.2302	*0.1304*	-1.4164	*0.0927*	0

**PMS2**	1.0811	*0.7581*	**1.6842**	*0.0721*	**1.9255**	***0.0141***	**1.7533**	***0.0469***	**1.8982**	***0.0264***	**1.6072**	***0.0318***	**4**
POLB	**-1.5886**	*0.1199*	-1.1075	*0.3515*	1.0996	*0.7008*	-1.0445	*0.6807*	1.0346	*0.6658*	-1.1183	*0.4368*	0
POLD3	**-1.6395**	*0.1344*	-1.1485	*0.4468*	1.0863	*0.7306*	-1.0865	*0.6363*	-1.0042	*0.9791*	-1.1548	*0.4479*	0
PRKDC	**-1.9241**	***0.0106***	-1.3065	*0.1697*	-1.1151	*0.6037*	-1.241	*0.1468*	-1.1366	*0.2418*	-1.3386	*0.0531*	1
RAD17	1.4489	*0.0713*	1.4358	*0.1169*	**1.7191**	***0.0035***	1.1003	*0.705*	1.1317	*0.5728*	1.3471	*0.1419*	1
RAD18	**-1.8985**	***0.0275***	-1.2519	***0.0308***	-1.0852	*0.6865*	-1.1972	***0.02***	-1.1101	*0.083*	-1.3006	***0.0209***	1
RAD21	**-1.5716**	*0.0876*	-1.0803	*0.5966*	1.0766	*0.7699*	-1.0282	*0.8256*	1.0509	*0.5888*	-1.1085	*0.4726*	0
RAD23A	-1.4199	*0.2207*	1.0736	*0.7283*	1.266	*0.2838*	1.115	*0.4882*	1.2165	*0.2634*	1.0355	*0.8206*	0
RAD23B	**-1.6342**	*0.0576*	1.0079	*0.9696*	1.1095	*0.3449*	1.0382	*0.8205*	1.1227	*0.5105*	-1.0643	*0.5959*	0
RAD50	**-2.0101**	***0.0214***	-1.3928	***0.0079***	-1.1628	*0.5299*	-1.3226	***0.0071***	-1.2224	***0.0125***	-1.4163	***0.0218***	1
RAD51	**-1.6867**	***0.0302***	-1.1298	*0.2798*	1.0319	*0.875*	-1.0799	*0.2538*	1.0066	*0.9076*	-1.1657	*0.0746*	1
RAD51B	**-1.533**	*0.1298*	-1.033	*0.6816*	1.1287	*0.6311*	1.0147	*0.8751*	1.0916	*0.301*	-1.0653	*0.6214*	0
RAD51C	**-1.8888**	***0.0393***	-1.3249	***0.0371***	-1.1183	*0.7019*	-1.2564	*0.0654*	-1.1591	*0.1653*	-1.3452	*0.0989*	1
RAD51D	-1.3064	*0.3306*	1.3051	*0.5481*	1.3598	*0.2548*	1.3219	*0.4689*	1.4312	*0.3768*	1.1875	*0.5912*	0
RAD52	**-1.5643**	*0.1258*	1.0145	*0.9435*	1.1623	*0.3556*	1.0491	*0.7768*	1.1368	*0.4719*	-1.0393	*0.7916*	0
RAD54B	1.0616	*0.6711*	1.0358	*0.7593*	1.297	*0.2019*	-1.2681	*0.1951*	-1.2139	*0.342*	-1.0156	*0.9152*	0
RAD54L	**-1.8586**	***0.0244***	-1.2978	*0.1693*	-1.0997	*0.6983*	-1.2265	*0.2267*	-1.1337	*0.3117*	-1.3194	*0.1139*	1
**REV1**	**2.1806**	***0.0012***	**2.1343**	***0.0002***	**2.6328**	***0.0075***	**1.6106**	***0.005***	**1.6835**	***0.0132***	**2.0146**	***0.002***	**6**
RFC1	**-1.8213**	***0.0425***	-1.3193	*0.2222*	-1.0692	*0.799*	-1.2381	*0.2735*	-1.1381	*0.3727*	-1.3144	*0.183*	1
RPA1	**-1.5496**	*0.1135*	-1.0613	*0.6765*	1.1255	*0.6028*	-1.0077	*0.9464*	1.0744	*0.3943*	-1.0828	*0.5464*	0
RPA2	**-2.6667**	***0.0234***	1.0994	*0.5006*	1.3591	***0.0457***	-1.1873	*0.2619*	-1.1319	*0.3187*	1.046	*0.6576*	1
RPA3	**-1.63**	*0.0827*	-1.1201	*0.1781*	1.0598	*0.8179*	-1.0667	*0.3389*	1.0144	*0.8164*	-1.1443	*0.289*	0
RRM2B	-1.0467	*0.7896*	-1.0656	*0.7386*	1.1669	*0.3634*	-1.4075	*0.1671*	-1.3495	*0.2216*	-1.1271	*0.5156*	0

SHPRH	-1.2054	*0.1306*	-1.2358	*0.0725*	1.0181	*0.9029*	**-1.6217**	***0.0196***	**-1.5438**	***0.0348***	-1.2965	*0.0527*	2
SIRT1	1.3356	*0.0681*	1.2888	***0.0402***	**1.6512**	*0.0568*	-1.0194	*0.883*	1.04	*0.7959*	1.2377	*0.1402*	0
SMUG1	**-3.3532**	***0.034***	**-2.6203**	*0.0856*	**-1.9811**	*0.1233*	**-2.4718**	*0.1016*	**-2.3332**	*0.1061*	**-2.55**	*0.0748*	1
**SUMO1**	**1.8373**	***0.0027***	**1.7862**	***0.0002***	**2.2501**	***0.0142***	1.3509	***0.0214***	1.4246	***0.0434***	**1.7005**	***0.0049***	**4**
TDG	**-2.4452**	***0.0028***	-1.4901	*0.1015*	-1.389	*0.1749*	-1.4416	*0.0636*	-1.3097	*0.139*	**-1.5973**	***0.0183***	2
TOP3A	-1.4839	*0.2639*	1.2848	*0.6031*	1.2586	*0.5739*	1.3376	*0.5525*	1.425	*0.492*	1.142	*0.7485*	0
TOP3B	**-1.546**	*0.2837*	1.0577	*0.9012*	1.2249	*0.6469*	1.1149	*0.8162*	1.2621	*0.6186*	1.0174	*0.9666*	0
TP53	-1.1506	*0.3673*	-1.1907	*0.3059*	1.0558	*0.7549*	**-1.5257**	*0.0651*	-1.455	*0.0584*	-1.2359	*0.1898*	0
TP53BP1	-1.126	*0.3968*	-1.1378	*0.3573*	1.0622	*0.6864*	-1.4927	*0.0602*	-1.4402	*0.0816*	-1.2104	*0.2002*	0
TREX1	**-1.6964**	*0.2577*	-1.2308	*0.6037*	-1.0656	*0.8914*	-1.1579	*0.7105*	-1.097	*0.7911*	-1.2508	*0.5784*	0
UBE2A	1.0707	*0.3054*	1.0361	*0.7109*	1.3192	*0.0785*	-1.264	*0.1011*	-1.1933	*0.1322*	-1.0059	*0.9349*	0
UBE2B	**1.6132**	***0.0007***	**1.5716**	***0.0031***	**1.9936**	***0.0107***	1.1756	***0.0004***	1.2526	***0.0165***	1.4947	***0.0012***	3
UBE2N	1.3876	*0.0937*	1.3826	*0.1795*	**1.6491**	***0.0239***	1.0402	*0.8472*	1.0783	*0.6816*	1.2876	*0.1826*	1
UBE2V2	1.3999	***0.0334***	1.3553	*0.0513*	**1.7096**	*0.0572*	1.0225	*0.7572*	1.0868	*0.5135*	1.2926	*0.0866*	0
UNG	**-1.6451**	*0.1259*	-1.1404	*0.0555*	1.0402	*0.8951*	-1.0807	*0.438*	-1.0053	*0.9543*	-1.1616	*0.3824*	0
WRN	1.3319	***0.0246***	1.307	*0.0606*	**1.6005**	***0.0337***	-1.012	*0.9165*	1.0319	*0.8193*	1.2318	*0.0989*	1
XAB2	**-1.6759**	***0.0425***	-1.0368	*0.8521*	1.0372	*0.8629*	1.0007	*0.9964*	1.0911	*0.5578*	-1.1079	*0.4436*	1
XPA	**-1.5196**	*0.0884*	1.0224	*0.8815*	1.1468	*0.5964*	1.0646	*0.5847*	1.1711	*0.2923*	-1.0247	*0.8497*	0
XRCC1	**-1.9321**	***0.0347***	-1.2492	*0.408*	-1.0723	*0.8333*	-1.1999	*0.484*	-1.0682	*0.8097*	-1.2927	*0.3185*	1
XRCC2	-1.2231	*0.3562*	1.3025	*0.1854*	1.448	*0.0545*	1.3578	*0.1042*	1.4585	***0.0407***	1.2294	*0.0944*	0
XRCC4	**-1.6478**	*0.0895*	-1.1496	*0.1333*	1.0354	*0.8992*	-1.0911	*0.3574*	-1.0122	*0.8602*	-1.1692	*0.2987*	0
XRCC5	-1.2922	*0.2214*	1.2197	*0.2518*	1.3632	*0.088*	1.2649	*0.0676*	1.3682	***0.023***	1.1539	*0.0598*	0
XRCC6	-1.1706	*0.4969*	1.3286	*0.1276*	1.4681	*0.1366*	1.3803	***0.0188***	**1.5055**	***0.0116***	1.2606	*0.0728*	1

[a] 5HKGs: expression value based on the average of the five reference genes (ACTB, B2M, GAPDH, HPRT1, and RPLP0); [b]FC: fold-change; [c] S-score: each point is gained when a gene shows FC ⩾ 1.5 and P-value ⩽ 0.05 upon normalization to either one of the six reference genes; only genes with score ⩾ 4 are considered to be

significant; [d] Differentially expressed genes and values of FC ⩾ 1.5, P-value ⩽ 0.05, and score ⩾ 4 are highlighted in bold.

Table S3. Gene expression of 12 selected DNA repair genes individually normalized to six different reference genes in PM and CR0 cells upon exposure to 20 μM of $CdCl_2$ for 72 h.

Genes	ACTB		B2M		GAPDH		HPRT1		RPLP0		5HKGs[a]		*S*-score[c]
	FC[b]	*P*-value	FC	*P*-value	FC	*P*-value	FC	*P*-value	FC	*P*-value	FC	*P*-value	
**PM / PM+Cd**													
DUT	**-1.9321**	***0.0007***	-1.0446	*0.671*	**-1.8724**	***0.0002***	**-1.5488**	***0.0062***	-1.1456	*0.1505*	-1.4627	***0.0048***	3
GADD45A	1.0685	*0.0975*	**1.983**	***0.0013***	1.102	*0.0513*	1.3349	***0.0136***	**1.8019**	***0.0023***	1.4126	***0.0026***	2
GTF2H2	**-1.5229**	***0.0108***	1.2136	*0.1832*	-1.4741	***0.0081***	-1.2201	*0.1416*	1.1098	*0.3621*	-1.1522	*0.209*	1
**REV1**	**-2.0772**	***0.002***	-1.1171	*0.204*	**-2.0089**	***0.0003***	**-1.6563**	***0.0004***	-1.2256	***0.0104***	**-1.5671**	***0.0011***	**4**
SUMO1	**-1.6515**	***0.0201***	1.1195	*0.5109*	**-1.5945**	***0.015***	-1.32	*0.1222*	1.0284	*0.8396*	-1.247	*0.1645*	2
ABL1	-1.2851	***0.0023***	1.4456	***0.0142***	-1.2473	***0.0221***	-1.029	*0.7431*	1.3106	*0.0621*	1.0284	*0.6983*	0
MBD4	**-1.8561**	***0.0013***	-1.0014	*0.9913*	**-1.797**	***0.0009***	-1.4849	***0.0185***	-1.0978	*0.3942*	-1.403	***0.017***	2
H2AFX	1.3959	*0.1689*	**2.5811**	***0.0266***	1.4345	*0.179*	**1.7325**	*0.0974*	**2.3387**	***0.0355***	**1.838**	*0.0633*	2
**LIG3**	**-2.417**	***0.0002***	-1.2986	*0.0861*	**-2.342**	***0.0001***	**-1.9301**	***0.0022***	-1.4306	***0.0149***	**-1.8254**	***0.0013***	**4**
**PCNA**	**-2.0182**	***0.0002***	-1.0808	*0.3855*	**-1.955**	***0***	**-1.6079**	***0.0009***	-1.1928	*0.0641*	**-1.522**	***0.0005***	**4**
PMS2	**-1.841**	*0.0712*	1.0004	*0.9987*	**-1.7721**	*0.064*	-1.4711	*0.1918*	-1.0792	*0.7234*	-1.389	*0.2263*	0
NEIL1	-1.4775	*0.5751*	1.2181	*0.7406*	-1.3995	*0.6127*	-1.1804	*0.7968*	1.1822	*0.7663*	-1.1114	*0.865*	0
**CR0 / CR0+Cd**													
DUT	**-1.6254**	***0.0106***	-1.0868	*0.3386*	**-1.7315**	***0.0021***	-1.2152	*0.0558*	-1.245	*0.1605*	-1.3585	***0.0134***	2
GADD45A	1.2117	*0.1127*	**1.8112**	***0.0012***	1.1275	*0.383*	**1.6123**	***0.0057***	**1.5565**	***0.0482***	1.4407	***0.0178***	3
GTF2H2	-1.3797	***0.0334***	1.0847	*0.4216*	-1.4693	***0.0265***	-1.0336	*0.7247*	-1.0685	*0.6407*	-1.156	*0.1748*	0
REV1	-1.4473	***0.0057***	1.0374	*0.5981*	**-1.5411**	***0.013***	-1.0815	*0.1988*	-1.1124	*0.3003*	-1.2094	***0.019***	1
SUMO1	-1.4577	***0.0138***	1.0307	*0.6983*	**-1.5454**	***0.0063***	-1.0871	*0.1739*	-1.117	*0.2396*	-1.2156	***0.015***	1
**ABL1**	**1.5618**	***0.0182***	**2.3441**	***0.0016***	1.4696	*0.0568*	**2.087**	***0.0018***	**2.0088**	***0.0039***	**1.8649**	***0.0035***	**5**

MBD4	-1.4212	***0.0174***	1.0535	*0.5317*	**-1.5136**	***0.0138***	-1.0636	*0.4261*	-1.0953	*0.4599*	-1.1893	*0.065*	1
**H2AFX**	**9.8269**	***0.0276***	**14.379**	***0.0156***	**8.8726**	***0.0206***	**12.816**	***0.02***	**12.307**	***0.0306***	**11.457**	***0.0214***	**6**
**LIG3**	**3.2066**	***0.01***	**4.7982**	***0.0043***	**3.0263**	***0.0185***	**4.2758**	***0.0041***	**4.0999**	***0.0013***	**3.8223**	***0.0048***	**6**
PCNA	**-1.6128**	***0.001***	-1.072	*0.4242*	**-1.7169**	***0.0109***	-1.2063	*0.0609*	-1.2516	*0.158*	-1.3499	***0.0165***	2
**PMS2**	1.3262	*0.0575*	**1.9833**	***0.0007***	1.2316	*0.1236*	**1.7729**	***0.0036***	**1.7491**	***0.0286***	**1.5862**	***0.0082***	**4**
NEIL1	**1.7068**	*0.183*	**2.5406**	*0.0627*	**1.5877**	*0.2958*	**2.2543**	*0.0758*	**2.1582**	***0.0399***	**2.0173**	*0.1025*	1

[a] 5HKGs: expression value based on the average of the five reference genes (ACTB, B2M, GAPDH, HPRT1, and RPLP0); [b] FC: fold-change; [c] S-score: each point is gained when a gene shows FC ⩾ 1.5 and P-value ⩽ 0.05 upon normalization to either one of the six reference genes; only genes with score ⩾ 4 are considered to be significant; [d] Differentially expressed genes and values of FC ⩾ 1.5, P-value ⩽ 0.05, and score ⩾ 4 are highlighted in bold.

Table S4. Expression of 12 differentially expressed DNA repair genes in lung cancer patients obtained from the online database and grouped by patients' smoking status.

Gene	Non-smokers vs. smoker		Non-smoker vs. ex-smoker (≤15 years)		Non-smoker vs. ex-smoker (>15 years)		Smoker vs. ex-smoker (≤15 years)		Smoker vs. ex-smoker (>15 years)		Ex-smoker (≤15 years) vs. ex-smoker (>15 years)	
	Exp.	Sig.	Exp.	Sig.	Exp.	Sig.	Exp.	Sig.	Exp.	Sig.	Exp.	Sig.
	**Lung Adenocarcinoma (TCGA, Provisional)**											
ABL1	↑	*	↑	*	-	ns	-	ns	-	ns	-	ns
DUT	-	ns	-	ns	-	ns	↓	*	-	ns	-	ns
GADD45A	-	ns	-	ns	-	ns	-	ns	-	ns	-	ns
GTF2H2	-	ns	-	ns	-	ns	-	ns	-	ns	-	ns
H2AFX	↑	****	↑	*	-	ns	↓	*	↓	****	↓	****
LIG3	↑	**	↑	*	-	ns	-	ns	↓	**	↓	*
MBD4	-	ns	-	ns	-	ns	-	ns	-	ns	-	ns
NEIL1	↓	**	↓	*	-	ns	-	ns	↑	**	↑	*
PCNA	↑	***	↑	*	-	ns	↓	*	↓	***	↓	*
PMS2	-	ns	-	ns	-	ns	-	ns	↓	**	↓	**
REV1	↓	**	-	ns	-	ns	-	ns	↑	****	↑	*
SUMO1	-	ns	-	ns	-	ns	-	ns	-	ns	-	ns
	**Lung Squamous Cell Carcinoma (TCGA, Provisional)**											
ABL1	-	ns	-	ns	-	ns	-	ns	-	ns	-	ns
DUT	-	ns	-	ns	-	ns	↓	*	-	ns	-	ns
GADD45A	-	ns	-	ns	-	ns	-	ns	-	ns	↑	*
GTF2H2	-	ns	-	ns	-	ns	-	ns	-	ns	-	ns
H2AFX	-	ns	-	ns	-	ns	-	ns	↓	**	↓	*
LIG3	↑	*	↑	*	-	ns	-	ns	-	ns	-	ns
MBD4	↑	**	↑	**	↑	**	-	ns	-	ns	-	ns
NEIL1	-	ns	-	ns	-	ns	-	ns	↓	**	↓	*

**PCNA**	-	ns	-	ns	-	ns	-	ns	-	ns	-	ns
**PMS2**	-	ns	-	ns	-	ns	-	ns	-	ns	-	ns
**REV1**	↑	*	↑	*	-	ns	-	ns	-	ns	-	ns
**SUMO1**	-	ns	-	ns	-	ns	-	ns	-	ns	-	ns

For lung adenocarcinoma dataset: non-smokers (n=76), smoker (n=119), ex-smoker (⩽15 years) (n=135), and ex-smoker (>15 years) (n=169). For lung squamous cell carcinoma dataset: non-smokers (n=18), smoker (n=133), ex-smoker (⩽15 years) (n=250), and ex-smoker (>15 years) (n=83). Exp.: mean expression level; Sig.: significance level (only P ⩽ 0.05 is considered statistically significant); ↑: up-regulated; ↓: down-regulated; ns: not significant; *P ⩽ 0.05; **P ⩽ 0.01; ***P ⩽ 0.001; **** P ⩽ 0.0001.

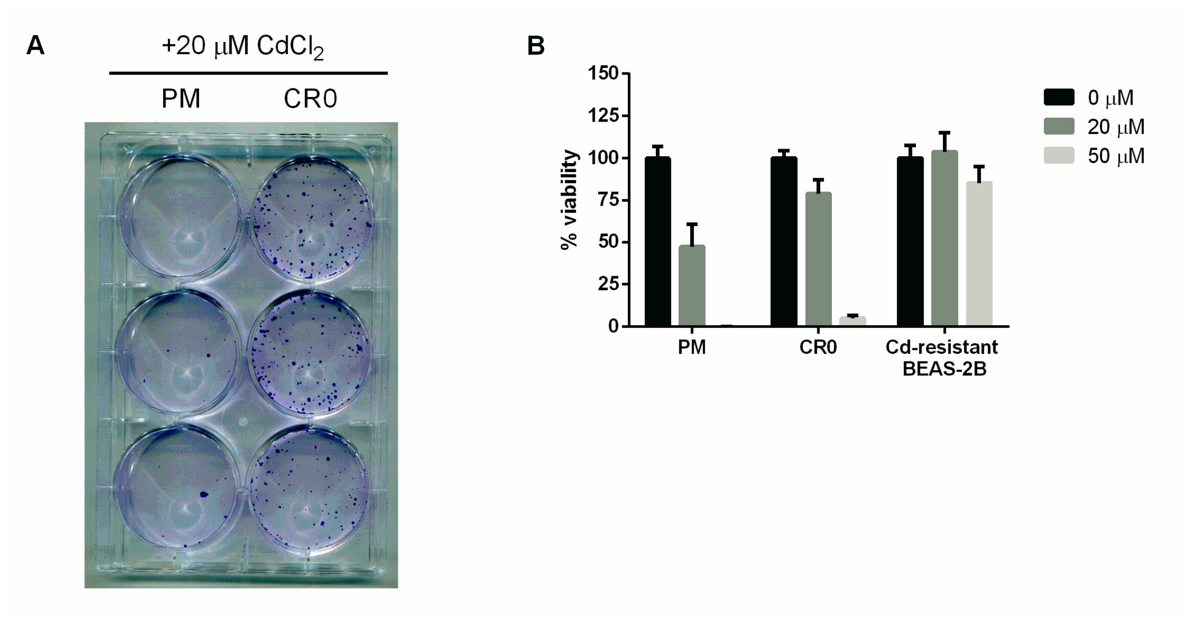

Figure S1: Determination of the Cd-resistance of the post-chronic Cd-exposed cells. (**A**) Morphology foci of PM and CR0 cells treated with 20 μM of $CdCl_2$ and grown for seven days were observed using a light microscope and photographed. (**B**) Cell viability of PM, CR0, and Cd-resistance BEAS-2B cells (maintained in 20 μM $CdCl_2$) exposed to different concentrations of $CdCl_2$ for 72 h was measured by MTS assay (absorbance at 495 nm).

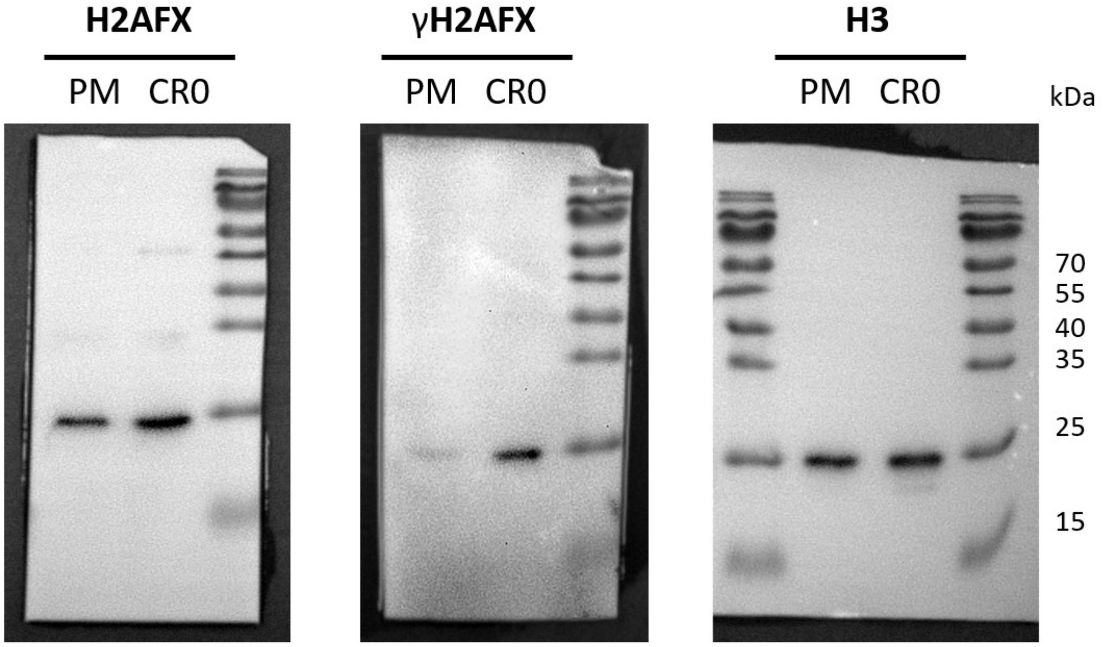

Figure S2: Western blot of histones extracted from CR0 and PM cells probed with H2AFX (1:1000, A11361, ABClonal), γH2AFX (1:1000, AP0099, ABClonal), or H3 (1:2000, 4499, CST) antibody. Marker used was PageRuler™ Prestained Protein Ladder (Thermo Scientific, USA).

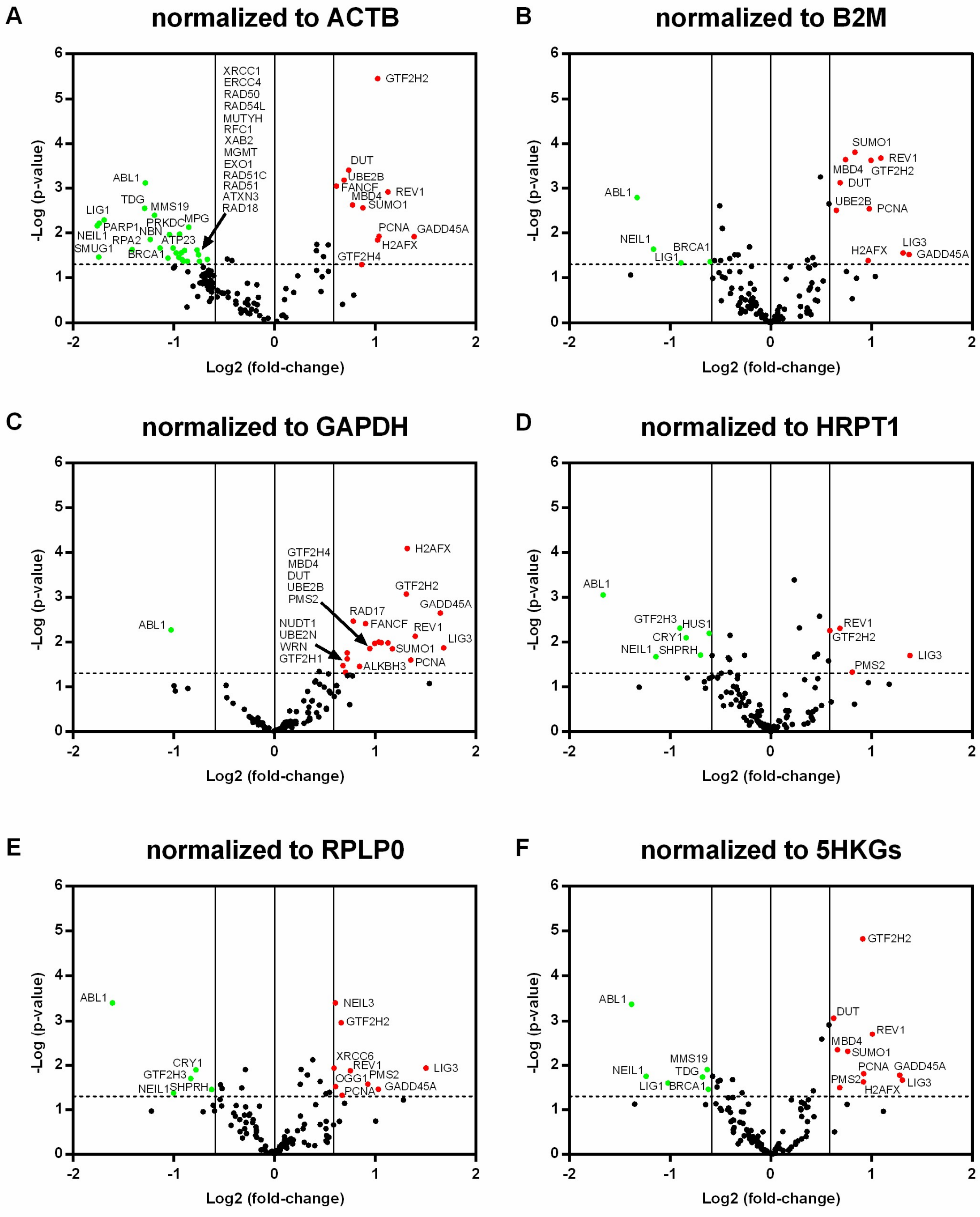

Figure S3: Volcano plots of 117 DNA repair gene expression profile of CR0 over PM cells when individually normalized to six different references genes: (**A**) *ACTB*, (**B**) *B2M*, (**C**) *GAPDH*, (**D**) *HPRT1*, (**E**) *PRLP0*, and (**F**) 5HKGs (based on the average of *ACTB*, *B2M*, *GAPDH*, *HPRT1*, and *PRLP0*). Significantly differentially expressed genes (P ≤0.05 and fold-change ≥1.5) were highlighted in green (down-regulated) or red (up-regulated).

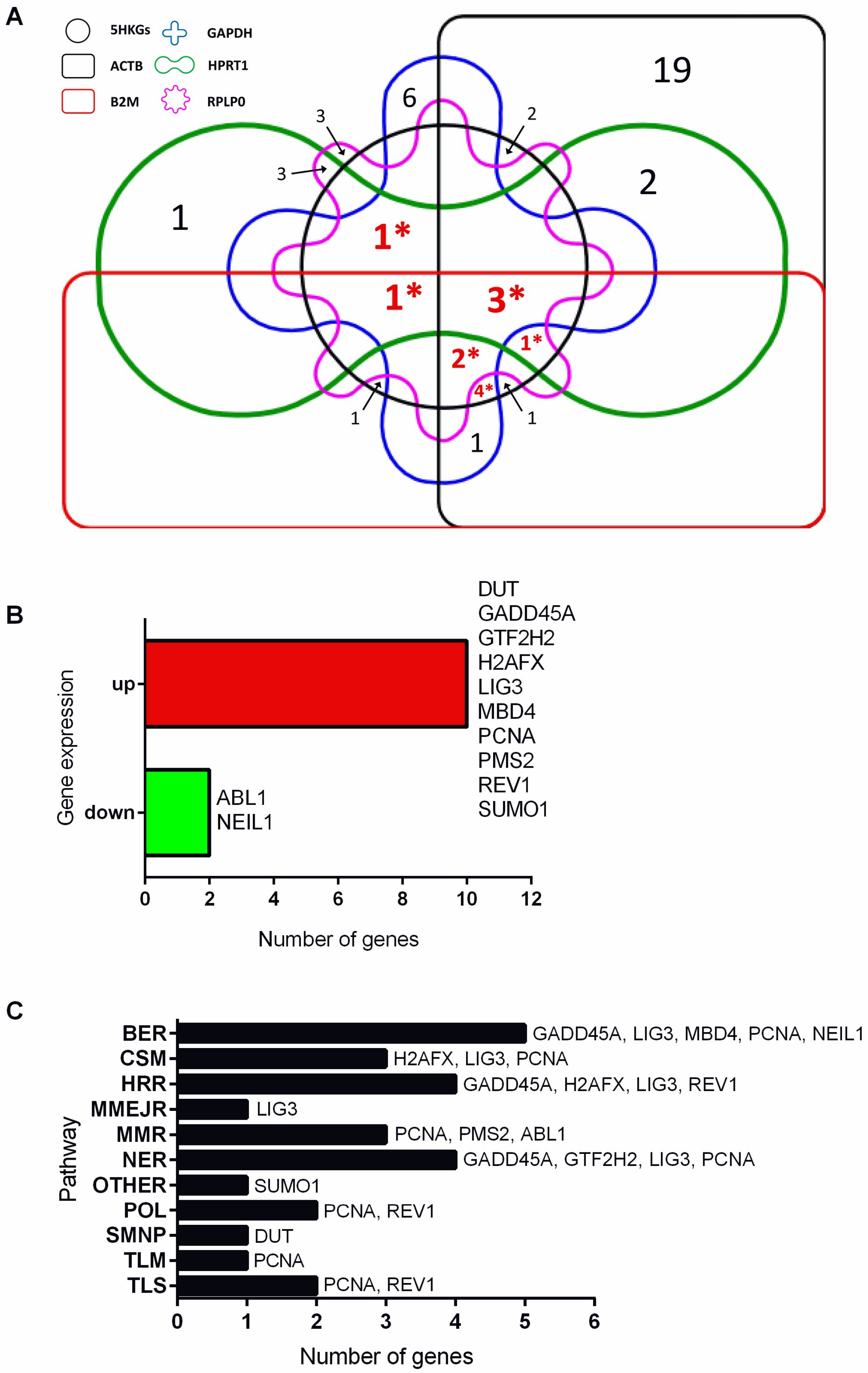
A
5HKGs
GAPDH
ACTB
HPRT1
B2M
RPLP0
19
6
3
3
2
2
1
1*
1*
3*
1*
2*
4*
1
1
1
B
DUT
GADD45A
GTF2H2
H2AFX
LIG3
MBD4
PCNA
PMS2
REV1
SUMO1
ABL1
NEIL1
Gene expression
up
down
0 2 4 6 8 10 12
Number of genes
C
BER GADD45A, LIG3, MBD4, PCNA, NEIL1
CSM H2AFX, LIG3, PCNA
HRR GADD45A, H2AFX, LIG3, REV1
MMEJR LIG3
MMR PCNA, PMS2, ABL1
NER GADD45A, GTF2H2, LIG3, PCNA
OTHER SUMO1
POL PCNA, REV1
SMNP DUT
TLM PCNA
TLS PCNA, REV1
Pathway
0 1 2 3 4 5 6
Number of genes

Figure S4: Differentially expressed DNA repair-related genes of the CR0 cells over control PM cells. (**A**) Venn-diagram shows the number of genes that were significantly up- or down-regulated ($P \leq 0.05$ and fold-change ≥1.5 when normalized to either one of the six reference genes; numbers in overlapping set ≥4 (*s*-score ≥4) are highlighted in red and asterisk. (**B**) Number of genes with significantly up- or down-regulated expression in CR0 cells. (**C**) The 12 differentially expressed DNA repair genes and their associated DNA repair pathways.

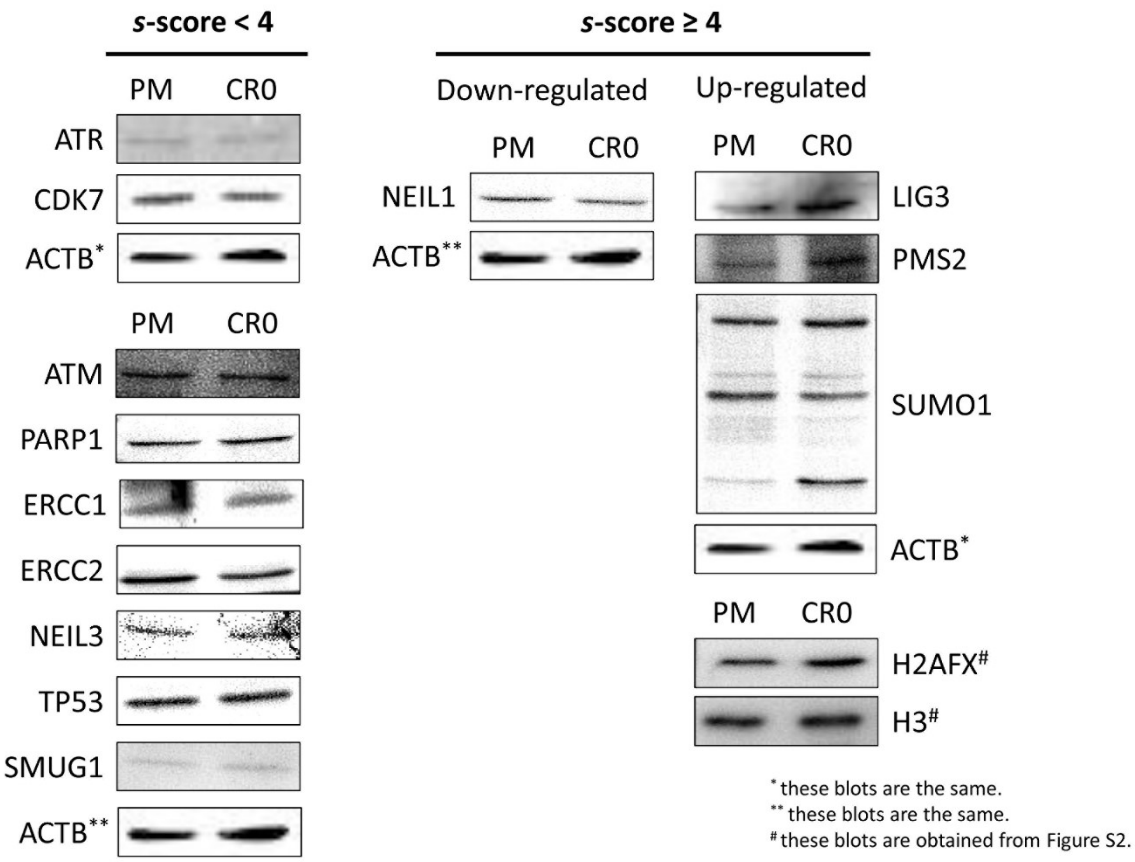

Figure S5: Protein expressions of selected DNA repair genes were determined by western immunoblotting. Antibodies were purchased from Santa Cruz Biotechnology: ATR (sc-515173); ATM (sc-377293); CDK7 (sc-365075); ERCC1 (sc-17809); LIG3 (sc-390922); NEIL1 (sc-271164); NEIL3 (sc-393703); PARP1 (sc-8007); PMS2 (sc-25315); SMUG1 (sc-377370); SUMO1 (sc-5308), GeneTex: ERCC2 (GTX108948); TP53 (GTX70214), Sigma-Aldrich: ACTB (A5441), ABClonal: H2AFX (A11361), and Cell Signaling Technology: H3 (4499). The H3 was used as a control for H2AFX whereas ACTB was used for the rest of the proteins. Experiments were carried out using different batches of samples and thus the multiple ACTB data.

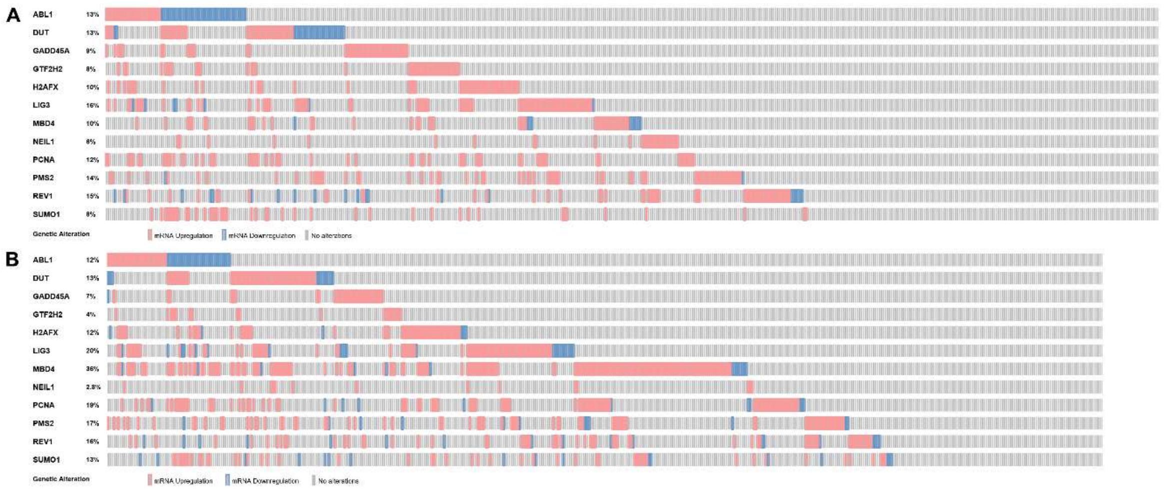

Figure S6: Expression of the 12 differentially expressed DNA repair genes in lung cancer patients obtained from TCGA datasets and visualized using cBioportal database. (**A**) Data from 517 lung adenocarcinoma samples (TCGA, provisional). (**B**) Data from 501 lung squamous cell carcinoma samples (TCGA, provisional). Each bar represents individual patient: bars highlighted in red represent up-regulated gene expression (z-score >+1.5) whereas those highlighted in blue represent down-regulated gene expression (z-score <-1.5); grey bars represent no significant differences in gene expression between the diseased and healthy control samples (z-score is between -1.5 and +1.5). For both datasets, the mRNA expression z-score threshold is set at ±1.5.
